# Spatial Proteomics and Epigenomics Reveal Cell Morphology and Epigenetic Gene Regulation in Human Hippocampus

**DOI:** 10.21203/rs.3.rs-10632086/v1

**Published:** 2026-09-07

**Authors:** Yang Xiao, Graham Su, Yuqi Tan, Nima Asaad, Yanxiang Deng, Tianyu Li, Yao Lu, Dongjoo Kim, Gorazd B. Rosoklija, Archibald Enninful, Zhiliang Bai, Yang Liu, Cheick Sissoko, Madeline Mariani, Tingting Wu, Phi Nguyen, Huiyi Liang, Adrienne Santiago, Andrew J. Dwork, René Hen, Garry P. Nolan, J. John Mann, Sai Ma, Kam W. Leong, Maura B. Dupont, Rong Fan

**Affiliations:** 1 Department of Biomedical Engineering, Columbia University, New York, NY 10027, USA; 2 Department of Pathology, University of Michigan Medical School, Ann Arbor, MI, 48109, USA; 3 Department of Biomedical Engineering, Yale University, New Haven, CT 06510, USA; 4 Yale Stem Cell Center, Yale School of Medicine, New Haven, CT 06520, USA; 5 Microbiology and Immunology Department, School of Medicine, Stanford University, Stanford, CA 95304 USA; 6 Department of Genetics and Genomic Sciences, Icahn School of Medicine at Mount Sinai, New York, NY 10029, USA; 7 Icahn Genomics Institute, Icahn School of Medicine at Mount Sinai, New York, NY 10029 USA; 8 Department of Pathology and Laboratory Medicine, Epigenetics Institute, Perelman School of Medicine, University of Pennsylvania, Philadelphia, PA 19104 USA; 9 Department of Psychiatry, Columbia University, New York, NY, 10032 USA; 10 Division of Molecular Imaging and Neuropathology, New York State Psychiatric Institute, New York, NY 10032 USA; 11 Macedonian Academy of Sciences & Arts, Skopje, North Macedonia; 12 Herbert Irving Comprehensive Cancer Center, Columbia University Irving Medical Center, New York, NY 10032, USA; 13 Division of Systems Neuroscience, New York State Psychiatric Institute, New York, NY 10032, USA; 14 Department of Pathology and Cell Biology, Columbia University, New York, NY 10032, USA; 15 Department of Neuroscience, Columbia University, New York, NY 10032, USA; 16 Department of Pharmacology, Columbia University, New York, NY 10032, USA; 17 Department of Pathology, School of Medicine, Stanford University, Stanford, CA 95304, USA; 18 Department of Radiology, Columbia University, New York, NY 10032 USA; 19 Department of Pathology, Yale University School of Medicine, New Haven, CT 06520 USA; 20 Human and Translational Immunology Program, Yale School of Medicine, New Haven, CT 06520, USA

**Keywords:** spatial omics, spatial epigenomics, spatial transcriptomics, spatial proteomics, human hippocampus

## Abstract

We present the first spatial tri-omic (proteomic, transcriptomic, and epigenomic) taxonomy of human hippocampus, addressing two key challenges in single-cell and spatial transcriptomics: visualizing cell morphology and epigenetic gene regulation *in situ*. By designing and multiplex imaging a novel panel of 25 protein targets, we generated the first large scale spatial proteomics map of the hippocampus at single cell resolution (330 nanometers). Through integrated spatial proteomic and transcriptomic analyses, we identified canonical hippocampal subfields and distinct cell types within the dentate gyrus and cornu Ammonis (CA). RNA synthesis rates were the highest in the CA pyramidal neurons than any other hippocampal subfields. To map gene regulation, we applied state-of-art spatial ATAC-seq to identify open chromatin regions and cis-regulatory elements, revealing how chromatin landscapes differ across hippocampal subfields. These regional differences help explain why certain neurons are more excitable and recruited for circuit formation, as their chromatin is open and responsive to transcription factors. Together, these approaches created the most comprehensive spatial atlas of the human hippocampus to date, consisting of over 1.6 million single cells from 16 individuals. This study demonstrates the use of spatial proteomics and epigenomics to investigate the molecular neuropathology of human brain in both healthy and diseased states.

## Introduction

The human hippocampus is essential for memory formation and spatial navigation and is critical in the limbic–cortical–striatal–pallidal–thalamic circuits that regulate mood, emotions, and learning. The hippocampus contains structurally distinct subfields, including the dentate gyrus (DG) and cornu Ammonis (CA) regions CA1, CA2, and CA3 ^1^, and shows significant cellular and molecular diversity ^2^. A comprehensive understanding of the hippocampus’s cellular and molecular landscape is crucial for studying normal brain functions and the mechanisms underlying various neurological disorders.

The cellular taxonomy of the human brain has primarily been investigated through two methodologies: single-cell analysis and spatial transcriptomics. Previous single-cell studies have provided a cell type-specific RNA database of the human hippocampus ^3–7^, but these data lack spatial context due to the dissociation involved in single-cell extraction from postmortem brain tissues. Dissociation disrupts cell-cell contacts and cell integrity, leading to the loss of neuronal and glial processes. Single-nucleus RNA (snRNA) sequencing from isolated nuclei also fails to capture transcriptomic information from the cytosol and neuropil, which constitute the majority of neuronal volume and are vital for synaptic activity. These components are crucial in the pathogenesis of neuropsychiatric disorders ^8,9^. Lack of spatial information mean that cellular subtypes and function cannot be mapped to their original microenvironments. For example, within the hippocampus’s trisynaptic circuit, granule neurons in the DG project to pyramidal neurons in CA3, which then relay signals to pyramidal neurons in CA1. The perforant path from the entorhinal cortex and parahippocampal gyrus to the DG, and projections from CA1 to the subiculum and fornix, are integral to this circuit ^10^. The precise connections are critical, but current understanding is hampered by the dearth of spatial data.

Recent spatial studies based on cytoplasmic mRNA capture indicate that there is considerably cellular diversity in the human prefrontal cortex ^11^ and mouse brain ^12–14^. Further, layer-specific gene profiles have shown that dysregulation of gene expression is associated with schizophrenia and autism spectrum disorders ^11^. Previous studies have not, however, fully elucidated the complexity of human hippocampus morphology, function, and epigenetic molecular characteristics ^15,16^.

In this study, we present the first spatial multi-omic atlas of the adult human hippocampus based on proteomic, transcriptomic, and epigenomic profiling. We evaluated 17 hippocampi from 9 neurotypical donors and 8 donors with major depressive disorder (MDD) (Figure 1A) (Table 1). The subjects had a mean age of 44 ± 12 years, with 15 males and 2 females. RNA quality consistently high across all hippocampal samples, with a mean RIN of 7.58 ± 0.88. The hippocampus is essential for understanding MDD due to its pivotal role in the brain’s mood and cognition circuits ^17,18^. Abnormally small hippocampal volumes ^19,20^ and altered neuronal connectivity ^21^ are linked to MDD, especially in untreated adolescents. These structural changes as well as aberrant interactions between the hippocampal DG and emotion-regulation areas like the ventrolateral prefrontal cortex are indicative of disruptions in critical neural pathways in patients ^22^. Additionally, chronic stress, common in severe MDD, leads to reductions in apical dendrites of hippocampal pyramidal neurons and disrupted synaptic connections in rodents ^23^.

We established a new experimental and computational workflow in spatial proteomics for neuroscience, which includes a custom-designed panel of 25 protein imaging targets to generate high-resolution cellular maps that included 1.6 million cells in human hippocampus. Importantly, this spatial proteomics approach preserves the tissue morphology and original cell organization, ensuring precise mapping of protein localization within the natural context of the tissue architecture. This dataset enables downstream bioinformatic study of cell-cell distance, microenvironment, protein-protein relationship, and protein-mRNA correlation. Spatial proteomic data offered unprecedented resolution of cellular morphology at the single cell level, with a larger detection area of tissues than spatial transcriptomics. Integration with single-cell and spatial transcriptomics allowed us to quantify the relative abundances of each cell type in hippocampal subfields and revealed variable RNA levels in MDD hippocampi compared to healthy control samples. RNA velocity analysis showed high inter-subject heterogeneity particularly in CA pyramidal neurons that was not observed in controls, suggesting that cell-type and gene-specific transcriptomic instability are characteristic of MDD. Genome-wide chromatin accessibility data at 50 μm resolution were obtained using our state-of-art spatial-ATAC-seq technology ^24^. For the first time, the epigenome of the human hippocampus was profiled with spatial contexts. Hippocampal subfield-specific transcription factors were identified based on open chromatin read peaks and motif sequence. Spatial ATAC-seq revealed the epigenetic regulation of different cell types in the hippocampus and showed that the chromatin states in granule neurons are more readily accessible for cFOS binding, indicating a primed state for memory formation. Together, we introduce and exemplify the use of spatial proteomics and spatial epigenomics in neuroscience research. This work provides a multi-omic atlas of human hippocampus for the research community. Our study highlights the importance of preserving spatial context in neurological research and underscores the potential of using spatial multi-omics to bridge the gap between traditional single-cell analyses and a more comprehensive, integrated understandings of brain function and disease.

## Results

### Single-cell taxonomy of human hippocampus by spatial proteomics

The primary aim of this study was to comprehensively characterize the neuronal, glial, immune, and stromal compartments within the architectural landscape of the human hippocampus using proteomic, transcriptomic, and epigenetic techniques (Figure 1C). To minimize confounding factors, we specifically selected postmortem human brain samples from cases of sudden death, ensuring the availability of comprehensive medical records and clear toxicology profiles. Medical, psychiatric, and psychosocial histories of all subjects (including controls) were determined through chart reviews and psychological autopsy ^25^, with appropriate written informed consent obtained.

We first performed 25-plex Co-Detection by Indexing (CODEX) ^26^ of hippocampal tissue from nine neurotypical controls and seven MDD patients (Figure 1D, Table 1). The 10-μm thick brain tissue sections were stained with a panel of 24 antibodies and 1 cell nuclei marker, sequentially imaged, processed, and systematically quantified (Figure 2A-C) employing methodologies previously reported ^27^. Distinct cell types, including neurons, astrocytes, oligodendrocytes, and microglia, were identified and visualized based on spatial coordinates (Figure 2D-E). The utilization of postsynaptic density protein 95 (PSD95) and Neurofilament-L (NFL) markers facilitated the demarcation of major dendrite-enriched and axon-enriched regions, respectively (Figure 2C). Following the quantification of protein marker expression, we used unsupervised clustering to identify six major cell types: neurons, astrocytes, oligodendrocytes, microglia, vascular cells, and immune cells in the CTRL01 neurotypical hippocampus sample (Figure 2F). In one slice of the CTRL01 sample, a total of 82,009 cells were identified and analyzed systematically (Table S1). Cell type-specific markers, such as NeuN and BDNF for neurons and OLIG2 for oligodendrocytes, were used to annotate the clusters (Figure 2G).

We observed variable expression percentages of the apolipoprotein APOE among different glial cell types. While previous studies suggested APOE^+^ cells are primarily astrocytes and, occasionally, microglia ^28^, we found APOE expression in astrocytes (35.96%), microglia (13.30%), oligodendrocytes (23.79%), and vascular cells (18.04%) (Figure 2H). The percentage of APOE^+^ oligodendrocytes was slightly lower than their total population percentage (28.54%).

We next overlaid the distribution of cell types onto their original spatial coordinates to characterize the hippocampal neuroanatomy structures (Figure 2I). The DG stratum granulosum was distinguished by the exclusive presence of small and densely packed granule neurons (Figure 2F, 2I). The DG stratum moleculare was enriched in dendrites (PSD95^+^) and separated from the CA by the vestigial hippocampal sulcus. The DG polymorphic layer united the granular layer to CA4 and was enriched in axons (Neurofilament-L^+^) of granular neurons. From the ventricular cavity toward the vestigial hippocampal sulcus, key layers of the CA were identified including the stratum pyramidale, molecular zone, and stratum oriens (Figure 2F, I). The DG stratum granulosum has mostly neurons, whereas the CA molecular zone is composed mostly of oligodendrocytes (Figure 2J). The rest of the regions have a heterogenous mixture of different cell types. The DG stratum granulosum has the highest neuron-to-glia ratio (14.95) of all hippocampal regions (Figure 2K). The diverse compositions of cell types suggest that the hippocampus consists of spatially heterogeneous subfields with organized pathways important for neural processing and synaptic connectivity.

### Multi-cellular neighborhoods and cell-cell distance

When we expanded our analyses to all samples, nine neurotypical control samples and seven MDD samples, we identified eight cell types (Figure 3A). Crucially, no significant batch effects were observed (Figure 3B). In addition to the six cell types identified from CTRL01, we identified choroid plexus epithelial cells and leptomeningeal cells when evaluating all samples. The choroid plexus epithelial cells were exclusive to CTRL04 (8.96%) and CTRL05 (4.61%), whereas leptomeningeal cells were identified in MDD01 (2.73%), MDD04 (5.11%), MDD07 (2.92%), CTRL02 (3.48%), CTRL04 (0.75%), CTRL05 (0.44%), CTRL07 (5.35%), and CTRL08 (0.33%) (Figure 3C and Table S1). The observed distribution of cell types aligned with the canonical anatomical regions of the brain (Figure 3D), validating our spatially resolved proteomic approach.

To investigate the spatial organization of these cell types, we examined cellular neighborhoods (CNs) of the hippocampus by identifying similar cell-type enrichment patterns using a nearest neighbor-based approaches.^29^ We identified nine distinct cellular neighborhoods (Figure 3E–F). Two CNs stand out as particularly enriched with vascular, immune, and choroid plexus epithelial cells: CN0 (Astrocyte Immune) and CN4 (ChPx_Epi Immune). Potential differential functionality likely arises due to the presence of astrocytes in CN4 but not in CN0. CN2 (DG Neuron) is enriched for granule neurons and has a pattern consistent with the DG stratum granulosum (Figure 3F). CN3 (CA Neuron) is enriched for both neurons and astrocytes, mirroring the patterns observed in the CA. Collectively, these findings highlight the power of CODEX imaging and neighborhood analysis to identify conserved brain regions that both consistent the gross anatomical knowledge and also discover finer molecular regional nuisances, pinpointing functionally distinct microenvironments within the intricate landscape of the brain.

In subjects with MDD, enduring priming of microglia has been reported (Rahimian et al., 2022; Wang et al., 2022). In our study, we observed that the distances between microglia and neurons, as well as between microglia themselves, were closer in MDD hippocampi than in control hippocampi (Figure 3G). This observation suggests that microglia may migrate toward neurons in MDD brains, contributing to neuronal inflammation. Understanding the specific contributions of these interactions in the context of MDD could shed light on the intricate interplay between the immune system and the neurobiology of depression.

### Spatial transcriptomic heterogeneity in the human hippocampus subfields

Spatial proteomics defines the cell types and phenotypes at single cell level, whereas spatial transcriptomics provides an unbiased molecular readout to identify the gene programs and pathways underlying those phenotypes. Integrating the two modalities enables discovery of mechanisms that cannot be inferred from a predefined protein panel alone. We used the DBiT-seq technology ^30^ to profile mRNAs in the hippocampi of staged flash-frozen postmortem human brains from six neurotypical controls and six MDD patients (Table 1, Figure 1A-C, Figure S1). Each hippocampal tissue section, with the DG positioned in the center, measured approximately 1 x 1 cm^2^ and was 10 μm in thickness. These sections were spatially barcoded (50 x 50 μm^2^ in pixel size, 2500 spots in 5 x 5 mm^2^ sequencing area), followed by cDNA synthesis and sequencing library generation as previously described ^31^ (Figure 1B). To confirm that the accuracy of regional annotation was free from effects of technical factors, we performed spatial mRNA-seq on biological replicates of three brains.

The resulting transcriptomic atlas revealed regional cellular heterogeneity in the subfields of the DG and CA (Figure 4A-D, Figure S2A-C). Based on a control brain (CTRL04), we examined gene expression in each cluster and annotated each cluster with its regional location. In total, unsupervised clustering of mRNA profiles identified seven hippocampal sub-regions (37,500 pixels in total): 1) GCL; 2) pyramidal neuron region of CA; 3) hilus region that contained inhibitory neurons; 4) molecular layer, polymorphic layer, and hilus; 5) white matter; 6) vasculature, immune cells, and other cells; and 7) choroid plexus (Figure S2D). Using genes highly expressed in each cluster, the signaling pathways active in each subfield were assessed (Figure 4E-F). *NEUROD2* and *GRIN2A* were expressed in all neurons, whereas *PROX1* was expressed by granule neurons and *SCN3B* was highly expressed in pyramidal neurons of the CA (Figure 4G-I, Figure S2A). *SST* was expressed in one subtype of inhibitory neurons (Figure 4J). Astrocytes expressing *NDRG2* and *AQP4* were enriched in the molecular layer of the DG and white matter, but not in the DG granule cell layer (GCL) (Figure 4K-L). Oligodendrocytes, which were identified by expression of *MOBP*, which encodes a myelin sheath protein, were located in the white matter (Figure 4M). Microglia were sparsely detected in all regions (Figure S2B). Epithelial cells, which express high levels of *CLDN5*, were abundant in the choroid plexus (Figure 4N). The cell-type assignments were confirmed by neuropathology analyses of adjacent Nissl-stained sections. Additionally, we compared the spatial proteome data and spatial transcriptome data of the samples. *RBFOX3* mRNA and the NeuN protein that it encodes were both detected in all neurons (Figure 4O-P). PSD95, predominantly located in the dendrites of excitatory synapses, marked the dendrite-enriched polymorphic layer, molecular layer, and hilus (Figure 4Q). The distribution of AQP4 highlighted the astrocyte-enriched region (Figure 4R); this region was also marked by expression of *NDRG2* and *AQP4* mRNAs (Figure 4K-L).

### Cell type deconvolution by integration with reference single-nuclei RNA datasets

Comprehensive mapping of the abundance of distinct cell types with high sensitivity and accuracy in human hippocampus region is challenging because cell types and densities vary substantially in subfields. Unsupervised clustering of spatial transcriptomes and snRNA-seq data mapped the pixels corresponding to the major cell types but did not allow determination of the cell-type compositions of each pixel (Figure 1B, S3A-C). The machine learning computational pipeline cell2location accounts for latent variables in techniques, batches, and varying cell density across locations ^13^. Using cell2location, we first derived the reference gene signatures from a previously reported snRNA-seq dataset (n= 219,058 nuclei, 69 cell subtypes) ^3^ and then computed the relative cell-type abundances in our spatial spots. The resulting maps were consistent with anatomical hippocampus subfields (Figure 4T-U, S3E-F). For instance, granule cells were most abundant in the DG GCL as predicted. Similarly, pyramidal neurons mapped onto the CA3 region (Figure 4U, S3F-G). Astrocytes and oligodendrocytes were preferentially distributed in the white matter and DG molecular zone. We detected five subtypes of inhibitory neurons (InN SST ADAMTS12, InN NR2F2 ANO2, InN NR2F2 SLC17A8, InN LAMP5 NMBR, InN LAMP5 CHST9) previously described in hippocampus ^3,32^. These showed diverse distribution patterns in our study (Figure S3D, S4). As expected, vascular and leptomeningeal cells were detected in choroid plexus, and pericytes were found close to vasculature (Figure 4T-U). We validated cell-type annotations in all representative regions by neuropathological review on adjacent Nissl-stained sections following published morphology criteria ^33^ (Figure 4A).

We performed non-negative matrix factorization of cell-abundance estimates from cell2location to detect co-localized cell types, although it should be noted that signature marker genes greatly overlapped. Nineteen distinct cellular compartments with varying cell compositions were detected (Figure S3D). Four types of oligodendrocytes (Oligo CPXM2 KANK4, Oligo OPALIN SLC5A11, Oligo OPALIN LAMA2, Oligo OPALIN LINC01098) co-localized (Figure S3D). In contrast, inhibitory neuron subtypes (InN SST ADAMTS12, InN LAMP5 CHST9, InN NR2F2 SLC17A8, InN NR2F2 ANO2) did not co-localize (Figure S4). Inhibitory neuron subtypes spread out in a mutually exclusive distribution pattern, whereas oligodendrocyte subtypes shared a niche. We mapped the most abundant cell types in each pixel in a representative MDD sample (Figure S3E-F) and observed a consistent pattern of each cell type distribution in the hippocampus subfields. The fine-grained cell maps of 12 human hippocampi (Figure S3G) could be used as a resource for understanding the co-occurring cell type combinations in brain function and disease.

### RNA velocity analyses reveal region-specific cell states involved in synapse plasticity

To understand the kinetics of cell states in the human hippocampus, we computed RNA velocities based on the sequencing counts of nascent pre-mRNAs and mature (i.e., spliced) mRNAs, using the state-of-the-art method scVelo ^34,35^. RNA velocity, the time derivative of the gene expression state, was computed based on differential equations that model the kinetics of transcription, splicing, and degradation of mRNA molecules. The balance between unspliced and spliced mRNAs is predictive of cellular states, where high levels of pre-mRNA indicate high levels of gene expression, and an excess of spliced mRNA indicates low levels gene expression. The velocity profiles of each gene in each single cell reveal information about RNA turnover with high accuracy and throughput.

Spatially variable patterns in RNA expression (Figure 5A-F) and RNA velocity (Figure 5G-L) were observed for genes involved in neurotransmitter transport and synapse plasticity regulation. For instance, the glutamate transporter *SLC1A2* was expressed at high levels and its RNA velocity score was high in the DG stratum moleculare region (Figure 5A, G). This represents a pattern of high expression with rapid turnover. The mRNAs encoding two GABA transporters, *SLC6A1* and *SLC6A7*, had complementary mRNA expression distributions: *SLC6A1* was expressed at high levels in the white matter region, but *SLC6A7* was predominantly detected in the neuron-enriched CA region (Figure 5B-C). Regions with high mRNA levels of *SLC6A7* displayed a low turnover of RNA synthesis (Figure 5C, 5I). This represents another correlation pattern of high expression with slow turnover. Furthermore, there were considerable regional differences in genes associated with synaptic vesicle trafficking and cytoskeleton remodeling such as *CLSTN2*, *PCLO*, and *TTLL7* (Figure 5D-F, J-L). This spatial atlas of RNA velocity will provide a valuable resource for identifying molecules that change with age or disease, which could potentially be quantified using magnetic resonance imaging or positron emission tomography.

### RNA dynamics differ in hippocampal regions and in brains of healthy subjects and those with MDD

We next used the spatial mRNA atlas to provide insights into region- and disease-specific gene expression. Using UMAP to visualize the unsupervised clustering results, we found spatial pixels clustered primarily based on regional cell identity (Figure 5M) rather than disease state (Figure 5N). The key gene marker in each subfield in the integrated dataset was the same as in individual samples (Figure 5M), suggesting that the gene markers specifying region identity are the same in healthy subjects and those with MDD. In other words, cell states, not cell types, are altered in MDD.

We examined the differentially expressed gene profiles in MDD versus control subjects in different sub-regions (Figure 5O). In the GCL region, *HSP90B1*, which encodes a molecular chaperone, was expressed at higher levels in control than MDD samples (avg_log2FC = 5.70, adj_p_val = 5.23E-05) as was SRPK2, which encodes a presynaptic zone regulatory factor (avg_log2FC = 3.69, adj_p_val *=* 3.75E-05). Also in the GCL, genes involved with synaptic plasticity (*PCLO*, avg_log_2_FC = 38.35, adj_p_val = 1.80E-42), vesicle trafficking (*NBEA*, avg_log_2_FC = 37.03, adj_p_val = 4.17E-50), and sodium transport (*SLC4A7*, avg_log_2_FC = 22.51, adj_p_val = 2.09E-59) were expressed at higher levels in MDD samples than controls. In the pyramidal neurons in the CA, there were lower levels of expression of genes involved in ion transport (*SYT1*, avg_log_2_FC = 79.44, adj_p_val = 1.23E-39), synaptic plasticity (*YWHAB*, avg_log_2_FC = 3.99, adj_p_val = 3.31E-85), and neuronal homeostasis and maintenance (*NNAT*, avg_log_2_FC = 17.26, adj_p_val = 9.06E-17) in MDD than control samples. Inhibitory neurons comprised less than 3% of total spots in our brain sections. Group comparison showed a higher expression of *SST* (avg_log_2_FC = 18.38, adj_p_val = 4.30E-6) and *NPY* (avg_log_2_FC = 33.69, adj_p_val = 6.16E-05) in controls. No significant differences in expression levels were observed for gene express in the vasculature (e.g., *CALD1*, *CLDN5, MYH11, TAGLN*) between MDD and control samples.

Next, we investigated how the cell states differed in hippocampal subfields from a perspective of RNA velocity. We first applied unsupervised clustering to spatial pre-mRNA and mature mRNA data (Figure S5A-B). In both control samples and MDD samples, cells with the highest RNA dynamics were detected in the CA, and dynamics were considerably lower in the DG GCL (Figure S5B). The distribution of the proportion of mature mRNA in MDD and in control samples (Figure 5P-Q) was significantly different (n_CTRL_=17500, n_MDD_=19740, Kolmogorov-Smirnov Test, p-value < 2e-16). The variance of the spliced mRNA proportion was significantly larger in MDD (Levene’s test, variance was 0.04 in MDD and 0.01 in CTRL, p-value < 2e-16) compared to controls, indicating greater dispersion in the MDD group. To further investigate the variables contributing to mature mRNA proportions, we applied a linear mixed-effects model that accounted for interactions between group, region, age, PMI, and within-sample correlation. In addition to group effects, the region of the pixel significantly influenced RNA dynamics. After adjusting for age, PMI, and within-sample correlation, the differences in the proportion of mature mRNA between the MDD and CTRL groups were as follows: 0.07 [95% CI: 0.06 – 0.08] in the GCL, 0.03 [95% CI: 0.025 – 0.038] in the ML_PL_H, 0.03 [95% CI: 0.02 – 0.05] in the Vas_Other, and 0.04 [95% CI: 0.03 – 0.05] in the WM. The increased variation in the MDD group may reflect clinical heterogeneity among subjects, such as differences in disease severity, stress exposure, benzodiazepine treatment, and brain changes in agonal stage.

We observed three types of transcriptomic stages, induction, repression, and steady state, by examining the velocity and expression of the most dynamic genes. Excess of pre-mRNA relative to the computed equilibrium rate indicated an induction stage. *BCAS1*, a gene encoding a myelin sheath protein in oligodendrocytes, was at the induction stage in white matter (Figure S5D). In spatial anatomical spots with high expression of *BCAS1*, this gene also had a high RNA velocity score (Figure S5D). *MAP1B*, a gene encoding a protein involved in neuron microtubule assembly, was detected in all three transcriptomic stages in a manner that depended on the spatial location. In most GCL and CA spots the RNA velocity score of *MAP1B* was close to zero, indicative of an mRNA in equilibrium, but some white matter and hilus spots had positive RNA velocity scores indicative of induction stage and others had negative RNA velocity scores indicative of repression (Figure S5E). The velocity and expression of *MAP1B* were highly heterogeneous in the sub-regions of hippocampus, suggesting some areas are undergoing dendrite outgrowth ^36^, while others are not.

Interestingly, certain genes, such as GRIN2A and NRGN, exhibited opposing trends in velocity and expression in specific regions (Figure S5F-G). NMDA receptors are involved in long-term synaptic potentiation, a key process in memory and learning ^37^. GRIN2A, which encodes a subunit of the N-methyl-D-aspartate (NMDA) receptor, showed high expression but low velocity in most granule cell layer (GCL) and CA regions (Figure S5G), suggesting an equilibrium state. In certain spots within the hilus and molecular layer, GRIN2A displayed positive velocity, indicative of induction. The differential spatial pattern of GRIN2A suggests distinct RNA states, leading us to hypothesize that GRIN2A may function differently depending on its spatial localization. One possible explanation is that the granule cells in dentate gyrus (GCL) requires a steady state of GRIN2A to support long-term potentiation, whereas the hilus may trigger a transient increase in GRIN2A expression only during spontaneous excitatory neurotransmission. This finding highlights the strength of spatial transcriptomics in dissecting cell states and their functional roles.

Next, we explored how region-specific RNA velocities differ between subjects with MDD and healthy controls. By examining the pre-mRNA to mRNA ratios of the 50 genes with the highest RNA velocity scores, we found that genes involved in synapse assembly (e.g., *CLSTN2, PCLO, SYNE2*) differed between MDD and control samples (Figure 5R–T, S5F-Q). *CLSTN2*, which encodes a calcium ion binding protein that regulates synapse assembly and synaptic transmission, showed significant differences in RNA dynamics. Linear regression analysis revealed that the slopes between the two groups were significantly different (slope difference was 0.43 [95%CI: 0.41 – 0.45]). More *CLSTN2* RNAs were in the induction stage in control samples compared to MDD samples (Figure 5R). In contrast, *SST* pre-mRNA was only detected in the MDD samples (Figure 5T). *SST* encodes a neuromodulator that is involved in the regulation of neurotransmitter release, neuronal excitability, and synaptic transmission. Expression of *PCLO*, which encodes a presynaptic cytomatrix protein, was previously reported to be associated with risk for MDD; PCLO is associated with temperamental traits, such as passivity, pessimism, and low self-esteem ^38^. *SYNE2*, which encodes a linker protein that connects the nucleus to the cytoskeleton, was previously found to be critical in retinal neuron development ^39^. In MDD samples *SYNE2* had a much wider distribution in velocities than observed in controls (Figure S5H-K). Genes coding for potassium ion channels (*KCNQ5*), a muscarinic cholinergic receptor (*CHRM3*), a glutamate transporter (*SLC1A2*), and inflammatory response factors (*IFI44L, IFI6*) also showed altered RNA velocities in MDD (Figure S5L-P). We did not observe significant difference between MDD and control samples in the dynamics of genes such as *BCAS1* and *RELN* (Figure S5Q-R). These results demonstrate that RNA velocity analyses provide a new perspective that will further our understanding of the molecular underpinnings of MDD. Future studies in a larger cohort will be needed to confirm these gene expression abnormalities in MDD.

### Spatially resolved chromatin accessibility profiling identifies region-specific epigenetic alterations in the MDD hippocampus

We detected region-specific alterations in gene expression and RNA dynamics within the hippocampi of individuals with MDD compared to neurotypical controls. To understand the underlying gene regulatory mechanisms, we utilized spatial-ATAC-seq ^24^ to profile chromatin accessibility in 13 biological samples collected from five control and five MDD subjects (Fig. 6E, Table 1). A spatial barcoding scheme was applied to tag accessible genomic loci with Tn5 transposition, thus achieving cell-level resolution, genome-wide mapping of chromatin accessibility in the tissue samples.

We calculated transcription factor motif scores to identify the key regulators of gene expression as described previously ^40^. The motif scores serve as a quantitative measure of the likelihood of binding of a transcription factor at each pixel. By comparing the spatial plots for mRNA, RNA velocity, and transcription factor motif scores, we identified several spatial patterns. CREB5, a member of the CREB/ATF family, is involved in diverse physiological processes including memory formation, synaptic plasticity, and long-term potentiation ^41^. *CREB5* RNA expression and RNA velocity scores were highly correlated in the fimbria (region annotated in Figure 4A) and glia cell-enriched CA region, but the transcription factor motif scores were high in the pyramidal neuron-enriched CA region and low in the fimbria (Figure 6A). This suggests that pyramidal neurons in CA were more excitable via CREB5-induced gene expression than were granule neurons in GCL, because the former cells were primed with open chromatin at CREB5 motifs.

Spatial ATAC-seq offered a way to visualize the differential excitability of neurons, detecting patterns that are not apparent in mRNA expression level. FOS, a transcription factor involved in synaptic plasticity ^42^, exhibited no distinct spatial pattern in mRNA expression (Figure 6B). Although *FOS* gene expression levels were low - consistent with the sparse numbers of *FOS*-expressing cells involved in memory formation ^43^, the high motif scores suggest that most granule neurons in GCL are competent to rapidly activate cFOS-driven transcription, facilitating the recruitment of neuronal ensembles for new memory encoding.

NFIA, a member of the nuclear factor I transcription factor family, regulates astrocyte activity, which is necessary for memory formation and neuronal transmission ^44^. This gene was highly expressed in the GCL region and moderately throughout the other regions of the tissue (Figure 6C). RNA velocity scores were lower in the GCL region, as were transcription factor motif scores, than in other areas of the hippocampus.

NFE2L1, a nuclear factor, is essential for neuronal health and maintenance ^45^. *NFE2L1* mRNA expression was high in the CA but low in the GCL and white matter areas (Figure 6D). In contrast, RNA velocity was low throughout the entire tissue. The NFE2L1 motif score was also very low across the tissue. It was previously reported that some post-synaptic neurons are more excitable than their neighbors ^46^. Our approach offers an epigenetic explanation for this phenomenon: region-specific chromatin accessibility primes the cells to respond to different memory formation processes.

To understand the downstream transcription factor activity in MDD, we imputed the single-pixel gene expression levels using gene scores as previously described ^47^. A positive correlation between gene scores and motif scores indicates activator function, while a negative correlation suggests repressor activity. Multiple activating and repressing transcription factors were identified (Figure 6E). For example, ETV6, which regulates the maintenance of the developing vascular network in the brain ^48^, was identified as an activator and FOXG1 was identified as a repressor.

To further resolve region-specific patterns of accessibility within the hippocampus, we performed unsupervised clustering of the thirteen spatial-ATAC-seq samples. The GCL cluster (highlighted in circle, Figure 6F) was distinct and aligned with the anatomical GCL region identified by Nissl stain on an adjacent tissue section (Figure S6A-C). The other clusters were localized throughout the CA region, where inhibitory neurons, astrocytes, oligodendrocytes, and microglia were distributed at varying proportions (Figure SF6D-G). In MDD, transcription factors enriched in the GCL included MEF, FOXG1, RAR, ZNF, and IRX family members, while depleted transcription factors included several SOX and NFI family members (Figure 6G).

To examine the impact of genetic variants on the chromatin accessibility in MDD, we sought to identify chromatin peaks located close to single nucleotide polymorphisms (SNPs) associated with MDD, based on a genome-wide association meta-analysis ^49^. There were variations in chromatin accessibility between MDD and controls within genes known to be mutated in subjects with MDD that are related to neurotransmitter transport (e.g., *SLC6A7*), synaptic plasticity (e.g. *CAMK2A*), and ion transport (e.g. *CACNA2D1*) (Figure 6H). Notably, the polymorphism rs2240795 is located within the coding region of *SLC6A7* and most likely alters chromatin structure in this locus. The differential peaks in *CACNA2D1* presumably resulted from nearby polymorphisms rs37078 and rs10265730. *CAMK2A*, which encodes a protein crucial for memory processing, learning, and neuroplasticity in the brain (references), had distinct gene expression patterns in the GCL region of MDD compared to controls, as revealed by the spatial transcriptomics data. The differential peak near *CAMK2A* in the ATAC-seq data was located proximally to the rs61159377 polymorphism. Thus, our chromatin accessibility analysis revealed gene-specific epigenetic alterations in the GCL of MDD samples relative to controls and identified differentially regulated transcription factors and gene modules that are potential targets for therapeutic intervention.

## Discussion

We report here a comprehensive atlas of the human hippocampus generated using spatial multi-omics technologies. We demonstrated the best technological practices and interpretable findings of spatial proteomics, spatial transcriptomics and spatial epigenomics. Fresh frozen human brains stored for up to 18 years proved to be a reliable source of RNA and DNA for spatial assays. Our atlas provides functional insights about cell types and cell states in health and disease, revealing region-specific signatures in cellular and molecular landscapes in controls and MDD subjects. For the first time, the morphology and cell-cell interaction information were detected in the human hippocampus at single-cell level by spatial proteomics, comprising over 1.6 million cells. In addition to cell types, we also profiled the cell states by RNA dynamics in each hippocampal sub-field. Highly dynamic genes were identified, including *CLSTN2* and *SST*, that displayed a different RNA velocity in MDD than CTRL. Specifically, the pyramidal neurons in CA3 had higher RNA turnover in MDD versus control subjects. We demonstrated that RNA velocity is a predictor in cell state progression. An altered balance of pre-mRNAs and mature RNAs was observed more often, and the ratio was more variable in the MDD than control brains. The identified dynamic genes may serve as labeling targets for future *in vivo* neuroimaging or pharmacokinetics studies.

Previous studies suggested that early life stress impacts DNA methylation in the hippocampus and implicated epigenetic gene regulation induced by childhood adversity in MDD ^50,51^. Our spatial-ATAC-seq results identified the CREB family transcription factors as critical regulators of transcription in the CA and FOS/JUN family as most important in the DG. We compared the chromatin accessibility between MDD and control hippocampal samples and located the open chromatin regions that may regulate genes previously associated with MDD ^49^. Interestingly, spatial-ATAC-seq can be used to infer whether a transcription factor is an activator or a repressor, depending on the correlation of motif scores and gene scores. Our approach offers a method to study epigenetic regulation in tissue samples with high spatial precision. The analysis of motif scores provides a novel perspective on neuronal allocation and excitability in memory formation. We utilized spatial-ATAC-seq to visualize and predict regions where memory formation is likely to occur. Future work investigating other immediate early genes is expected to delineate the molecular mechanisms underlying how distinct neurons are recruited to a memory engram. Additionally, our integrated mRNA and epigenetic analyses have the potential to delineate the spatial organization of “cellular memory”, revealing, for example, whether repeated exposure to environmental stressors produces disproportional glial responses in psychiatric disease.

In conclusion, we have described our pioneering characterization of human hippocampal proteome, transcriptome and epigenome. We demonstrated that RNA velocity and chromatin accessibility data provide novel perspectives on the differences between healthy and MDD brains. The spatial omics resources generated in this study will serve as valuable references for researchers studying neurological diseases and for comparative studies between the human brain and brains of other organisms.

### Limitations of the Study

While our spatial multi-omic dataset provides significant insights into the cellular and transcriptional diversity of the human hippocampus, we did not incorporate spatial data registration with MRI images or investigate the anterior-posterior anatomical axis of the hippocampus. Unlike mice, where hippocampal structure is more uniform, the human hippocampus exhibits unique cellular organization. Much like fingerprints, no two human hippocampal sections align perfectly. Even within the same human brain, the left and right hippocampi may differ. Future studies will require high resolution MRI scans of the brain prior to spatial profiling to accurately register tissue sections to their anatomical planes.

Previous single-nucleus RNA sequencing (snRNA-seq) studies have demonstrated cell-type-specific gene expression differences between the posterior hippocampus—crucial for cognitive functions—and the anterior hippocampus, which is involved in mood regulation ^5^. Due to natural variations in sectioning orientation, obtaining identical coronal planes across different individuals is not feasible. In our study, we primarily used coronal sections taken around the mid-region of the hippocampus. This variability emphasizes the need for representative sampling along the entire anterior-posterior axis to construct a comprehensive functional atlas of the hippocampus.

We did not investigate the effects of brain lateralization. The dominance of brain hemispheres adds another layer of complexity; memory processing and other cognitive functions can differ between the left and right hippocampi. Unfortunately, our study lacked information regarding the brain dominance of the sampled individuals, which may influence hippocampal function and gene expression.

Although spatial proteomics provides single-cell resolution at approximately 300 nm, current spatial mRNA-seq and ATAC-seq technologies lack this level of resolution. Future advancements in spatial technologies will necessitate new designs for higher pixel resolution and larger mapping areas to enhance detection capabilities. Integrating data across different omic platforms will further improve the accuracy and depth of spatial profiles.

Current spatial biology findings lack depth and confidence in functional interpretation, due to limitation of the cohort size. Human samples inherently vary in demographics, processing, and biological heterogeneity. For instance, MDD is a complex disorder characterized by diverse clinical symptoms and heritability. Generalization of any spatial findings will require a much larger sample size. Data such as disease severity, age of onset, family history, stress exposure, body mass index, education level, and history of childhood adversity should also be collected and incorporated into future analyses. Additionally, we did not investigate the impacts of antidepressant treatment, cause of death, or postmortem interval on the transcriptomic changes in the brain. Systematic analysis of these factors, enabled by larger spatial and single-cell multi-omic datasets, will provide greater confidence and clarity in understanding their functional roles. Future studies incorporating larger cohorts will be essential for addressing these limitations.

## Methods

### Resource availability

#### Lead contact

Further information and requests for resources should be directed to and will be fulfilled by the lead contacts Drs. Rong Fan (rong.fan@yale.edu), Maura Boldrini Dupont (mb928@cumc.columbia.edu), and Kam Leong (kwl2121@columbia.edu).

#### Materials availability

This study did not generate new reagents.

### Experimental models and subject details

#### Postmortem human specimens

This study used de-identified human postmortem brain tissue from the Brain Collection of New York State Psychiatric Institute (NYSPI) (samples CTRL01, CTRL03 - 09, MDD01 - 08) and the National Institute of Mental Health’s Human Brain Collection Core (sample CTRL02) (Table 1). The NYSPI receives brain donations from North Macedonia, approved by the local Institutional Review Board. Autopsies were performed at the Institute for Forensic Medicine and Criminology at Ss. Cyril and Methodius University in Macedonia. Neuropsychiatric diagnoses, toxicology reports, and medical records were collected and preserved as previously described ^52^. In this study, the cohort included nine brains from subjects with no DSM-V psychiatric diagnosis and eight from subjects with MDD. Subjects were selected based on the following criteria: 1) sudden death with a short post mortem interval less than 48 hours; 2) no infectious disease (e.g., HIV, hepatitis, or untreated tuberculosis) or pre-existing neurological conditions; 3) no coma for 12 hours or more before death; and 4) high RNA integrity of the brain cortex tissue with RIN > 7.0. Neuropathological and toxicology examinations were performed after brain tissues were collected. Donor’s blood, urine, and hair were collected for toxicology tests. 192 of the most commonly prescribed or abused substances in Macedonia were tested by state-of-the-art liquid chromatography/quadrupole mass spectroscopy at the Biomarkers Laboratory Resources (Core Lab) at the Irving Institute for Clinical and Translational Research, Columbia University. Chart review and psychological autopsy ^25^ were performed to determine medical, psychiatric, and psychosocial history of each subject (including controls) with written informed consent from legally authorized representatives.

#### Brain tissue handling and preparation

We prepared flash frozen unfixed slices of the anterior-mid hippocampal region on the glass slides for spatial multi-omics assays. Briefly, coronal sections of the right brain hemisphere were serially cut at 20-mm intervals, held in shape with metal plates, and frozen in Freon. Tissue blocks were selected as close to the mid DG as possible (Figure 1A–B). We defined the anterior hippocampus (HPC) as the region from the rostral end of the hippocampus to the start of the lateral geniculate, and the mid HPC as the section from the lateral geniculate’s onset to its disappearance. The rest of HPC region was defined as posterior HPC. A coronal section of DG regions (~1 cm x 1 cm), identified by the same neuropathologist, were sliced at 10 μm and placed onto the center of the ultra-clean glass slide (Electron Microscopy Sciences, 63478-AS). All samples were stored at −80 °C before use.

#### RNA quality analyses

RIN number was assessed at the Herbert Irving Comprehensive Cancer Center at Columbia University. The RIN of brain cortex was determined and used to pre-screen the samples for inclusion. The RIN of hippocampus (Table 1) was determined on slices adjacent to the slice used for sequencing. Two fresh frozen tissue slices were placed in a 2-mL centrifuge tube, and RNA lysis buffer (Qiagen, #79216) and one 5-mm stainless steel bead (Qiagen, #69989) were added. The tissue was disrupted at 25 Hz for 4 minutes with a Qiagen TissueLyser II. Total RNA was isolated following the manufacturer’s instruction using miRNeasy Micro Kit (Qiagen, #1071023). RIN number was calculated based on the RNA electrophoresis using a Bioanalyzer (Agilent 2100) or a TapeStation System (Agilent 4200).

#### Immunohistology and imaging

Prior to conducting the spatial proteome profiling experiment using CODEX, we validated each primary antibody in human hippocampal tissue. Fresh frozen brain slices (10 μm) on glass slides were thawed at room temperature for 15-30 minutes before staining. Slices were fixed in 4% paraformaldehyde at room temperature for 15 minutes, followed by three washes with Dulbecco’s phosphate-buffered saline (DPBS). We used the peroxidase immunohistochemistry kit (Fisher, PI36000) following manufacturer’s instructions. Samples were first blocked with the peroxidase suppressor to quench endogenous peroxidase activity and then with 5% BSA solution with 0.05% Triton-X-100 for 2 hours at room temperature. After three washes with DPBS, the brain slices were incubated in primary antibody solution (1:100 dilution in blocking buffer) overnight at 4 °C. The Key Resources Table contains information antibodies used in this study. After a second set of washes, samples were stained with horseradish peroxidase-conjugated secondary antibody solution (1:500 dilution in blocking buffer) for 1 hour at room temperature. Freshly prepared DAB substrate working solution (Fisherbrand, 03000013) was used to stain slices for 2-5 minutes, depending on the background signal of the tissue observed in a light microscope. We counterstained the slides with hematoxylin solution for 1 minute and washed with deionized water for 15 minutes. Slides were mounted with mounting medium (ThermoFisher, 1859351) and set on a flat surface for at least 8 hours. All stained slices were scanned at 40x magnification by a slide scanner (Leica SCN400).

#### CODEX sample preparation

Frozen slides were placed in a closed container filled with Drierite adsorbents (Fisher Scientific, #23-116582) for 5 minutes to allow thawing and drying. Next, the slides were moved to a Coplin jar filled with acetone and incubated for 10 minutes at room temperature. After removal and drying for 2 minutes, the slides were placed in hydration buffer (Akoya, #7000017) and incubated for 2 minutes followed by incubation in fresh hydration buffer for 2 minutes. The slides were then submerged in 1.6% PFA (Electron Microscopy Sciences, #15710) for 10 minutes. After two more rounds of hydration buffer for 2 minutes each, the slides were moved to staining buffer (Akoya, #7000017) for at least 20 minutes. After this incubation, the slides were moved to staining buffer containing N/G/J/S Blockers (Akoya, #7000017) and antibodies and incubated at 4 °C overnight.

After staining, the slides were moved to staining buffer at room temperature for two rounds of incubation for 2 minutes each to remove excess antibody solution. Post-fixation was then performed for 10 minutes using 1.6% PFA. The slides were then rinsed three times with PBS before being placed in ice cold methanol for 5 minutes. After three more rinses with PBS, slides were incubated with fixative reagent (Akoya, #7000017) for 20 minutes at room temperature. The slides were then rinsed with PBS three times and placed in storage buffer (Akoya, #7000017) at 4 °C for up to 5 days.

#### CODEX multiplexed immunofluorescence imaging

For CODEX imaging, the slide was removed from the storage buffer and placed in PBS for 10 minutes. After drying with a tissue, a flow cell was placed on the slide and sealed. The assembled slide and flow cell was then placed in PhenoCycler Buffer made from 10X PhenoCycler Buffer & Additive (Akoya, #7000019) for at least 10 minutes prior to running the experiment.

A 96-well reporter plate (Akoya, #7000006) was prepared containing reporters corresponding to each barcoded antibody for each cycle with up to three reporters per cycle per well. The fluorescence reporters were mixed with 1X PhenoCycler Buffer and nuclear staining reagent (Akoya, #7000003), and assay reagent (Akoya, #7000003). The reporter plate was then sealed with an aluminum seal (Akoya, #7000007).

The reporter plate and the assembled slide and flow cell were placed into the CODEX machine, and the automated multiplexed imaging experiment was performed. During each cycle of imaging, there is a step for binding of the reporter, a step for imaging each of three fluorescent channels, and a step for stripping of the reporters to prepare for the next cycle. This is repeated until all the markers are imaged. Following the completion of the experiment, a qptiff image file containing individual antibody channels and DAPI channel are output.

#### CODEX data processing

DAPI-guided image stitching, drift compensation, deconvolution, and cycle concatenation were performed using the Akoya PhenoCycler software. Raw imaging data (qptiff, 377.442 nm/pixel for 20x CODEX) files and outputs from Akoya PhenoCycler software were first examined with QuPath software (https://qupath.github.io/) for inspection of staining qualities. Any markers that produced untenable patterns or a low signal-to-noise ratios were excluded from the ensuing analysis. We used SPACEc, an end-to-end CODEX analysis pipeline, to process all the CODEX data acquired.^29,53^ The qptiff files were first converted into tiff files for tissue detection (using the watershed algorithm) and cell segmentation. Whole-cell segmentation on the CODEX images was conducted using an implementation of CellSeg ^54^ with a pixel growth parameter set to 3 pixels. CellSeg segmentation outputs both the cell segmentation mask and the quantification of marker intensities within the masks. Subsequently, the signal originating from each individual cell was extracted. Each marker intensity was Z normalized separately for all cells within the dataset as previously described ^55^. This normalized the range of each marker as fluorescent intensities of each marker can depend on antibody staining strength and exposure times. Cells with no DAPI signal (artifacts) and expression of over 99% of all markers (noisy cells) were removed from the downstream analysis. To accomplish cell-type annotation, the Python package Scanpy (v1.9.3) was employed ^56^. The iterative utilization of the functions pp.neighbors (with n_neighbors = 10) and Leiden clustering generated unsupervised clusters of the data. These clusters were then subjected to manual annotation by experts using cell-type-specific markers. The masks were annotated using TissUUmaps (https://tissuumaps.github.io/).

#### Distance permutation and cell-cell interaction analysis

We performed cell distance analysis as previously described ^57^. Briefly, the Delaunay triangulation of cells was determined based on their Cartesian XY positions within each field of view using the default settings from the deldir R package. To identify interacting cells and their coordinates, we extracted the relevant information from the delsgs output of deldir. Subsequently, we computed the distances between cells connected by the edges of a Delaunay triangle in the two-dimensional space using the following formula: $\mathrm{Distance}=\sqrt{{\left(x_{1}-x_{2}\right)}^{2}+{\left(y_{1}-y_{2}\right)}^{2}}$. Cell-to-cell interactions within 100 μm were identified, resulting in 7,730,146 interactions. To establish a reference distribution of distances, we conducted 100 iterations of the triangulation calculation. In each iteration, the cells and their neighborhoods within each field of view were randomly reassigned to existing XY positions. We then calculated the average distance of cell-cell interactions for each field of view in each permutation. These resulting expected baseline distances were subsequently compared to the observed distances using a Wilcoxon test. The fold enrichment of distances between the observed data compared to the mean distances derived from the permutation test was determined as follows: $\log_{2}\;fold\;enrichment=\log_{2}\left(\frac{Observed\;mean}{Expected\;mean}\right)$. The log fold changes of distance for each pair of interactions where p values were less than 0.05 were used in analyses.

#### Cellular neighborhood analysis

In this study, we conducted a neighborhood analysis following a previously described method ^58^. Firstly, we utilized a window size of 40 nearest neighbors across the tissue cell-type maps. Within each window, we quantified the cells of each type, generating vectors representing cell counts. Subsequently, these vectors were subjected to clustering to identify commonly composed neighborhoods. After an extensive parameter search, we identified nine cellular neighborhoods that provided the most unique insights into the spatial organization and heterogeneity of cell types within the tissue. We used heatmap to visualize what cell types are uniquely enriched in each neighborhood.

#### Spatial context maps

Spatial context maps were created to understand the hierarchical relationship among neighborhoods as previously reported ^59^. We used window size of 45 nearest neighbors for each cell for creation of the composition vectors. Subsequently, we determined the combination that included the fewest neighborhoods that made up more than 85% of the neighborhoods within that window instead of clustering the vectors of neighbor compositions. This combination informs about prominent associations of neighborhoods in the window, a feature termed spatial context. We counted each combination and connected the most prevalent combinations together into a spatial context map.

#### Integration of spatial proteomes with snRNA-seq using MaxFuse

We performed MaxFuse to integrate our CODEX data with snRNA-seq data reported by Franjic *et al.* ^3^ as previously described ^60^. In brief, MaxFuse is a computational algorithm that matches cells across different modalities through fuzzy smoothing, canonical correlation co-embedding, iterative smoothing, and matching. Both shared and unshared features are utilized by MaxFuse to boost the accuracy of matched cells across RNA (thousands of features) to protein (<30 features). Joint embedding with all features preserves the cumulative information across many genes. We initiated MaxFuse with all gene features in snRNA-seq and CODEX data to construct a nearest-neighbor graph for each modality separately. Through meta cell construction (meta-cell size = 2), the sparsity in snRNA-seq data was dampened. MaxFuse smoothed the weakly linked features between snRNA-seq and CODEX data and then calculated a shared co-space to embed both modalities. For initial matching using shared features (<30), we assigned equal weights of 0.3 to both modalities. The matching process was iteratively refined by learning information from all features in both modalities. Cross-modal cell pairs were filtered using a threshold of 0.5 to obtain refined cell pairs of high quality. Subsequently, these refined cell pairs were employed to propagate the matching to the remaining cells. Poor matches between snRNA and CODEX cells were eliminated using a cutoff prediction score of less than 0.3. In our CODEX data, over 98% of cells were successfully matched to those in the snRNA-seq dataset.

#### DBiT-seq microfluidic device fabrication and assembly

The microfluidic device was fabricated with polydimethylsiloxane (PDMS) using soft lithography using the design previously described ^30,31^. The chrome photomask with 50-μm channel width were obtained from Front Range Photomasks. Master wafers for microwells were fabricated using SU-8 negative photoresist (Microchem, SU-8 2050) as follows: SU-8 was spin-coated on a clean silicon wafer at ~3000 rpm for 40 seconds to yield feature heights of 50 μm. We followed manufacturer’s (MicroChem) recommendations to perform SU-8 photo lithography, development, and baking. PDMS microfluidic chips were then fabricated via a replication molding process. The PDMS precursor was prepared by combining Sylgard 184 PDMS part A and part B (Dow Corning) at a 10:1 ratio. After stirring to mix, the mixture was poured into the master wafer, degassed for 30 minutes (or longer if there were bubbles), and cured at 75 °C for at least 2 hours. The solidified PDMS slab was cut out, peeled off, and the inlet and outlet holes were punched with a toggle press (Schmidt) to complete the fabrication. The inlet holes were 2 mm in diameter and held up to 10 μL of solution (depending on the PDMS height). A pair of microfluidic chips with the same location of inlets and outlets but orthogonal microfluidic channels in the center were fabricated as a complete set of devices for flow barcoding a tissue slide. For this, the PDMS slab was attached to the tissue section glass slides and a custom-designed acrylic clamp was used to firmly hold the PDMS against the tissue specimen to prevent leakage between microfluidic channels.

#### Fixation and permeabilization of tissue slices

The flash-frozen human hippocampus brain slides were taken from the −80 °C freezer and warmed at room temperature for about 10 minutes. PBS supplemented with RNase inhibitor (0.05 U/μL, Enymatics) was used to wash the tissue. Tissues were then fixed with 4% formaldehyde (Sigma) for 20 minutes at room temperature. The tissues were rinsed with PBS containing RNase inhibitor and then permeabilized with 1% Triton X-100 in PBS. Afterwards, the tissue slides were dipped in deionized water and then gently dried with nitrogen air.

#### Reverse transcription

To perform spatial barcoding of mRNAs for transcriptomic mapping, a PDMS reservoir was attached to the slide to encompass the tissue section. An reverse transcription mix made from 50 μL of RT buffer (5X, Maxima H Minus kit), 32.8 μL of RNase-free water, 1.6 μL RNase Inhibitor (Enzymatics), 3.1 μL SuperaseIn RNase Inhibitor (Ambion), 12.5 μL of dNTPs (10 mM, Thermo Fisher), 25 μL of reverse transcriptase (Thermo Fisher, #EP0751), and 100 μL of 0.5X PBS with RNase Inhibitor (0.05 U/μL, Enzymatics) was added to the reservoir to cover the entire tissue section. The slide and reservoir were incubated at room temperature for 30 minutes, then at 42 °C for another 90 minutes to allow the DNA oligonucleotides to bind to the mRNAs. To prevent evaporation, the slides were placed in a sealed humidified container. After this process, the slides were rinsed with NEB buffer 3.1 (New England Biolabs) supplemented with 1% RNase inhibitor (Enzymatics) for 10 minutes. The slides were then briefly dipped into a 50 mL Falcon tube containing RNase-free water to rinse off any remaining salts.

#### Barcoding spots with unique DNA barcodes

The first PDMS chip was carefully placed along the slide with the center channels covering the tissue region of interest. An acrylic clamp was used to ensure a tight fit of the PDMS with the slide to prevent any leakage between channels. A ligation mix of 69.5 μL of RNase free water, 27 μL of T4 DNA ligase buffer (New England Biolabs), 11 mL T4 DNA ligase (400 U/μL, New England Biolabs), 2.2 μL RNase inhibitor (40 U/μL, Enzymatics), 0.7 μL SuperaseIn RNase Inhibitor (20 U/μL, Ambion), 5.4 μL of 5% Triton X-100 in PBS was added. Into each channel was introduced 5 μL of solution containing 2 μL of the ligation mix, 2 μL of NEB buffer 3.1 (New England Biolabs), and 1 μL of DNA barcode A (25 μM) (Supplementary Table 2). A vacuum was used to pull the solution through the device, which was maintained at 37 °C, to saturate the channels to allow contact with the tissue. After 30 minutes, the microfluidic channels were washed with NEB buffer 3.1 (New England Biolabs). The first PDMS chip was then carefully removed and the slide was dipped in RNase-free water before drying with nitrogen air. Ligation of barcode B (Supplementary Table 2) was performed in the same fashion with the second PDMS chip with channels in the perpendicular direction to the tissue region of interest. The reaction was allowed to occur at 37 °C for 30 minutes and then the microfluidic channels were washed with 1X PBS supplemented with 0.1% Triton X-100 and 0.25% SUPERaseIn RNase Inhibitor for 10 minutes. Images showing the location of the microfluidic channels on the tissue slide can be taken during the flow step using light or dark field optical microscope (Thermo Fisher EVOS fl) before removal of the PDMS chips.

#### cDNA collection and purification

A PDMS reservoir was placed encompassing the tissue similar to the reverse transcription step to load lysis buffer onto the barcoded tissue region and to collect the barcoded cDNAs of interest. The lysis buffer consists of 2 mg/mL proteinase K (Thermo Fisher), 10 mM Tris, pH 8.0, 200 mM NaCl, 50 mM EDTA, and 2% SDS. The lysis was carried out at 55 °C for 2 hours and then collected and stored at −80 °C prior to use. A DNA Clean kit (Zymo Research) was used to extract the cDNAs from the lysis buffer. The collected cDNAs were resuspend it in 100 μL of RNase-free water. The cDNAs were then purified using streptavidin beads (Dynabeads MyOne Streptavidin C1 beads, Thermo Fisher, #65001). The beads (40 μL per slide) were first washed three times with 1X Binding and Washing (B&W) buffer (Thermo Fisher, refer to manufacturer’s manual of #65001) with 0.05% Tween-20 and then resuspended in 100 μL of 2X B&W buffer with 2 μL of SUPERaseIn RNase Inhibitor. The 100 μL of beads were then added to the cDNA and incubated for 60 minutes with gentle rotation. The beads with the captured cDNAs were then washed twice with 1X B&W buffer and once with 10 mM Tris buffer, pH 8, with 0.1% Tween-20.

#### Template switch and PCR amplification

The template switch reaction mix contains 44 mL of 5X Maxima RT buffer (Thermo Fisher), 44 mL of 20% Ficoll PM-400 solution (Sigma), 22 mL of 10 mM dNTPs each (Thermo Fisher), 5.5 mL of RNase Inhibitor (Enzymatics), 11 mL of Maxima H Minus Reverse Transcriptase (Thermo Fisher), and 5.5 mL of a template switch primer (100 mM). This was added to the washed beads. The sample was then incubated at room temperature for 30 minutes followed by an additional incubation at 42 °C for 90 minutes (both with gentle rotation). The beads were then rinsed with 10 mM Tris buffer, pH 8 and then with RNase-free water. A mixture of 110 mL Kapa HiFi HotStart Master Mix (Kapa Biosystems), 8.8 mL of 10 mM stocks of primers 1 and 2, and 92.4 mL of water was added to the beads, and PCR was performed using the following conditions: 95 °C for 3 mins, five cycles at 98 °C for 20 seconds, 65 °C for 45 seconds, and 72 °C for 3 minutes. The beads were then removed from the solution using a magnet. Evagreen (Biotium) was added to the supernatant at a 1:20 ratio, and a vial of the resultant solution was loaded into a BioRad thermocycler to perform a second PCR step with an initial incubation at 95 °C for 3 minutes, then 98 °C for 20 seconds, 65 °C for 20 seconds, and 72 °C for 3 minutes. The reaction was stopped when the fluorescence signal plateaued.

#### Amplicon purification, library preparation, and sequencing

The PCR product was purified using SPRIselect beads (Beckman Coulter) at 0.7X ratio. The cDNAs > 300 bp were then collected from the beads. If the cDNAs were less than 300 bp, they remained in the supernatant. The quality of amplicon was analyzed using a Tapestation system (Agilent). The resulting spatial 3’-end libraries were then built with a Nextera XT kit (Illumina) and sequenced using a NovaSeq 6000 sequencer (Novogene) at 150 x 150 pair-ended mode.

##### Sequence alignment and generation of gene expression matrix

To obtain transcriptomics data, the Read 2 received from sequencing was processed by extracting the unique molecular index (UMI), barcode A, and barcode B. The processed Read 1 was trimmed, mapped to the human genome (GRCh38), de-multiplexed, and annotated using the ST pipeline v1.8.1 ^61^, which generated the digital gene expression matrix for downstream analysis. The rows of the gene matrix correspond to pixels, defined by their location information (barcode A x barcode B), and columns correspond to genes. Examination of the UMI and number of detected genes showed comparable mRNA coverage among different samples (Figure S1).

#### Region annotations in the hippocampus

The Seurat package (v4.0.4) in R (v4.1.0) was used to assign region identities to each pixel in the unsupervised clustering results. First, normalization and variance stabilization of transcriptome data for each pixel with regularized negative binomial regression was performed using “SCTransform”, a module in Seurat. The process was similar to that widely used for scRNA-seq data normalization, with each pixel treated as a single cell. The expression matrix of all pixels was SCTransformed (“NormalizeData”, “ScaleData”, and “FindVariableFeatures”). Unsupervised clustering of spatial data identified distinct clusters, representing different cell layers in the hippocampus. We assigned a region identity to each cluster based on gene expression in spatial maps. For instance, a cluster with high levels of *PROX1* and *SEMA5A* was identified as GCL. To validate our annotations, two neuropathologists reviewed the adjacent slides with Nissl staining and identified cell types by their nuclei and cytoplasm morphologies following known criteria ^33^.

#### Differential expression analysis

Differentially expressed genes between samples or cell types were identified by using the FindMarkers function in Seurat. The following parameters were used: min.pct = 0.05, logfc.threshold = log(2). The top genes in each comparison group (CTRL_GCL vs. MDD_GCL; CTRL_CA vs. MDD_CA; CTRL_WM vs. MDD_WM; CTRL_ML vs. MDD_ML) were selected with a pre-filtering step (p_val_adj < 1e-30, pct.1 > 0.1, pct.2 > 0.1, avg_logFC>0.5 or <-0.5) to remove low count and insignificant genes, then were ranked by the absolute value of the log fold change. As the number of spots corresponding to the hilus region that contained inhibitory neurons in each group was low (<2% out of 2500 spots in each sample), we adapted the above cutoff as p_val_adj < 1e-7 in CTRL vs. MDD.

#### Single-cell mRNA data integration with spatial mRNA-seq

To assess key cell types in each pixel, we integrated the scRNA-seq reference data of six human hippocampus samples (GSE186538) ^3^ and our spatial transcriptome data in Seurat by specifying assay type as “scrna” or “spatial” in the metadata. Both the experimental and reference dataset were merged as one Seurat object, which was then normalized together by “NormalizeData”. “FindVariableFeatures” and “SelectIntegrationFeatures” were applied to find repeated variable features across the datasets. “FindIntegrationAnchors” and “IntegrateData” were applied to integrate the datasets together. Principal component analysis was performed using the “RunPCA” function. The first 30 principal components were used to construct the Shared Nearest Neighbor graph with “FindNeighbors” and clusters were identified with “FindClusters”. Co-clusters were mapped with UMAP ^62^. We grouped sub-clusters of the same cell type into one major type. For example, any cell type with Oligo in the name was mapped to the REF_Oligo major type.

#### Cell-type deconvolution with cell2location

To provide a high-resolution view of the cell-type composition in each pixel, we deconvoluted the spatial data using cell2location ^13^. First, we used the pre-annotated dataset of human hippocampus (GSE186538, n = 219,058 cells, 6 batches, 69 cell subtypes) described previously ^3^ as a reference to build the training model with all parameters set as default values. Then we ran cell2location (v0.5) on our raw spatial gene count matrices (N_cells_per_location=5, max_epochs=30000). Each pixel in the spatial data was resolved with probabilities of different cell types, corresponding to the absolute abundance of cells in that spot. The most abundant cell type in each region was determined based on the computed abundance scores of each of the 69 reference cell types.

#### RNA velocity analysis

We used scVelo to compute RNA dynamics and identify the cell state decision genes ^35^. scVelo requires no prior knowledge of the signature genes and it is not sensitive to library size variation. Briefly, we first prepared the loom files of the pre- and mature mRNAs from the sequencing bam file using Velocyto package (v0.17.16) using default settings ^34^. We then calculated cell dynamics (e.g., RNA splicing rates and degradation rates) using the scVelo package (v0.2.1) implemented in Python (v3.6) ^35^. Cell annotations with assigned region information were carried from the previous steps in Seurat. We used default settings in scVelo (mode=’stochastic’).

We first examined the distributions of spliced RNA proportions across different groups (Figure 5P–Q) and brain regions using histograms. Levene’s test and the Kolmogorov-Smirnov test were then conducted to assess the differences in variance and distribution between the MDD and CTRL groups, respectively. To further evaluate the differences in spliced RNA proportions between groups and regions, we fitted a linear mixed-effects model, which included an interaction term for group and region, age and PMI as confounding factors, and accounted for within-sample correlation. The model is specified as follows: Spliced RNA proportions = β_0_ + β_1_*group + β_2_*region + β_3_*(group*region) + β_4_;*age + β_5_*PMI + b + ε, where β_0_, β_1_, β_2_, β_3_, β_4_, and β_5_ are the fixed-effects coefficients, b represents the random effect that accounts for within-sample correlation, and ε denotes the error term. The difference in spliced RNA proportions between MDD and CTRL in GCL is 0.07 [95%CI: 0.06 – 0.08], in ML_PL_H is 0.03 [95%CI: 0.025 – 0.038], in Vas_Other is 0.03 [95%CI: 0.02 – 0.05], and in WM is 0.04 [95%CI: 0.03 – 0.05]), with the adjustment of age, PMI and within sample correlation. Additionally, to investigate the differences in the slopes of spliced versus unspliced CLSTN2 RNA between the MDD and CTRL groups, we employed a linear regression model for spliced CLSTN2 RNAs that included an interaction term for group and unspliced CLSTN2 RNA (Figure 5R). The difference in slopes between MDD and CTRL is 0.43 [95%CI: 0.41 – 0.45].

#### Tissue preparation for spatial-ATAC-seq

The fresh frozen human hippocampus brain slides were taken from the freezer and placed at room temperature for about 10 minutes to thaw. They were fixed with 0.2% formaldehyde (Sigma) in PBS for 5 minutes at room temperature. The tissue was quenched with 1.25 M glycine for 5 minutes at room temperature. The tissue was then washed with PBS, and the slides were dipped in deionized water and then gently dried with nitrogen air. The tissue was imaged for downstream alignment. Following the tissue scan, a PDMS reservoir was placed around the tissue. Lysis buffer (10 mM Tris-HCl, pH 7.4, 10 mM NaCl, 3 mM MgCl_2_, 0.1% Tween-20, 0.1% NP-40, 0.01% Digitonin, 1% BSA) diluted to 0.1X concentration using lysis dilution buffer (10 mM Tris-HCl, pH 7.4, 10 mM NaCl, 3 mM MgCl_2_, 1% BSA) was applied for 15 minutes at room temperature to permeabilize the tissue. The buffer was then removed, and wash buffer (10 mM Tris-HCl, pH 7.4, 10 mM NaCl, 3 mM MgCl_2_, 1% BSA, 0.1% Tween-20) was added to the reservoir for an incubation of 5 minutes. After removing the wash buffer, Transposition Mix (50 μL 2X tagmentation buffer, 33 μL PBS, 1 μL 10% Tween-20, 1 μL 1% digitonin, 5 μL loaded Tn5 transposome, and 10 μL nuclease-free water; the tagmentation buffer consists of 20 mM HEPES, 300 mM NaCl, 0.5 mM spermidine, 10 mM MgCl_2_, 1 tablet EDTA-free protease inhibitor cocktail in RNase-free water) was added, and the slide was incubated for 30 minutes at 37 °C. We then removed the tagmentation solution and stopped the reaction by a 5-minute incubation at room temperature in 40 mM EDTA. The tissue was washed with 1X NEBuffer 3.1 (New England Biolabs) and incubated for 5 minutes at room temperature. Lastly, we removed the wash solution and PDMS, dipped the slide in deionized water, and gently dried the slide with nitrogen air.

#### Unique *in situ* barcoding for spatial recognition

The initial PDMS chip was carefully positioned along the slide, aligning the target channel region with the tissue region of interest as was done for DBiT-seq. An acrylic clamp was used to ensure a secure fit of the PDMS with the slide, minimizing the risk of any inter-channel leakage. The ligation mix (72.4 μL RNase-free water, 27 μL T4 DNA ligase buffer (10X, New England Biolabs), 11 mL T4 DNA ligase (400 U/μL, New England Biolabs), and 5.4 μL of 5% Triton X-100 in PBS) was prepared. Each channel received 5 μL of a solution containing 2 μL of the ligation mix, 2 μL of NEB buffer 3.1 (1X, New England Biolabs), and 1 μL of DNA barcode A (25 μM). A vacuum was employed to draw the solution through the device, saturating the channels and enabling contact with the tissue. Sequences of barcodes A and B are given in Supplementary Table S2. After 30 minutes at 37 °C to ligated DNA barcode A to the reverse transcribed cDNA, the sample was washed with NEB buffer 3.1 (1X, New England Biolabs). Afterwards, the first PDMS chip was gently removed, and the slide was immersed in RNase-free water before being dried with nitrogen air.

The ligation of barcode B was conducted similarly to ligation of barcode A, but with the channels of the second PDMS chip oriented perpendicularly at the tissue region of interest. After 30 minutes at 37 °C, a combinatorial barcode A and B for each mRNA was obtained. The microfluidic channel was washed with 1X PBS supplemented with 0.1% Triton X-100 for 10 minutes. Images capturing the microfluidic channel locations on the tissue slide can be acquired during the flow step using a light or dark field optical microscope (Thermo Fisher EVOS fl) before peeling off the PDMS chips.

#### Lysis and library generation for spatial ATAC-seq

After barcoding the tissue, a small PDMS reservoir was placed on the tissue slide surrounding the tissue section. A lysis solution (50 mM Tris-HCl, pH 7.4, 1 mM EDTA, 1% SDS, 200 mM NaCl, 0.4 mg/mL proteinase K) was added to the reservoir. The slide was placed in a humidifying chamber and incubated at 58 °C for 2 hours. After incubation, the lysate was collected and incubated at 65 °C overnight with gentle rotation. The lysate was then purified with the DNA Clean & Concentrator kit (Zymo) and eluted with 21 μL with DNA Elution buffer (Zymo). The purified DNA was added to 2.5 μL each of i5 and i7 primers and 25 μL of 2X NEBNext Master Mix (New England Biolabs). The mix was then placed in a thermocycler and subjected to the following: 72 °C for 5 minutes, 98 °C for 30 seconds, then 5 cycles of 98 °C for 10 seconds, 63 °C for 30 seconds, and 72 °C for 1 minute. Aliquots of 5 μL of the PCR products were used for qPCR to determine additional cycles. The remaining 45 μL was then placed in the thermocycler and subjected to the following: 98 °C for 30 seconds, and then N cycles (with the value of N determined based on qPCR results) of 98 °C for 10 seconds, 63 °C for 30 seconds, and 72 °C for 1 minute. The products was purified using SPRIselect beads (Beckman Coulter) with incubation at room temperature for 10 minutes to bind the DNA. The beads were then washed twice while on the magnet with 200 μL of 80% ethanol each wash. Excess ethanol was removed, and beads were resuspended in resuspension buffer. The tube was mixed thoroughly and incubated at room temperature for 5-10 minutes to elute the DNA. The tube was then placed on a magnet and the supernatant was retrieved. All samples were stored at −20 °C prior to sequencing.

#### ATAC data integration, clustering, motif score calculation

The ATAC data containing read 1 with barcodes A and B for spatial identification and read 2 with genome sequences was aligned using the Cell Ranger ATAC software (10X Genomics, v2.1) to produce the processed BED-like fragment file. Human reference GRCh38 was used for alignment. Open chromatin region peaks were called from fragment files of individual samples using MACS2 peak caller ^63^. Peaks from all samples were then merged, and peaks overlapping with ENCODE blacklisted regions [https://sites.google.com/site/anshulkundaje/projects/blacklists] were removed. Peak summits extended by 250 bp on each side were defined as accessible regions. The number of fragments were counted in each accessible region to generate a peak by spot matrix using chromVAR ^40^.

The Integrative Genomics Viewer (v2.13.2) was used to compare differential peaks shown in the genomic tracks. We examined the gene tracks of the top 200 differentially expressed genes identified by spatial mRNA results and screened over 10,000 SNPs previously found in a genome-wide meta-analysis for MDD ^49^. Gene scores were calculated using a decay function by summing the signal centered around the gene promoter ^64^. To identify regulators, we computed accessibility scores for known transcription factor-binding motifs at the single-cell level using chromVAR ^40^. We then calculated the variation between MDD and controls by the transcription factor motif scores.

## Supplementary Material

This is a list of supplementary files associated with this preprint. Click to download.

- S.Table1CODEXcellcountspersample.csv
- S.Table2ListofPCROligoandDNABarcodeSequences.xlsx
- S.FiguresV7.pdf
- S.Movie1CTRL01SpatialProteome.mov

## Figures and Tables

**Figure 1.**
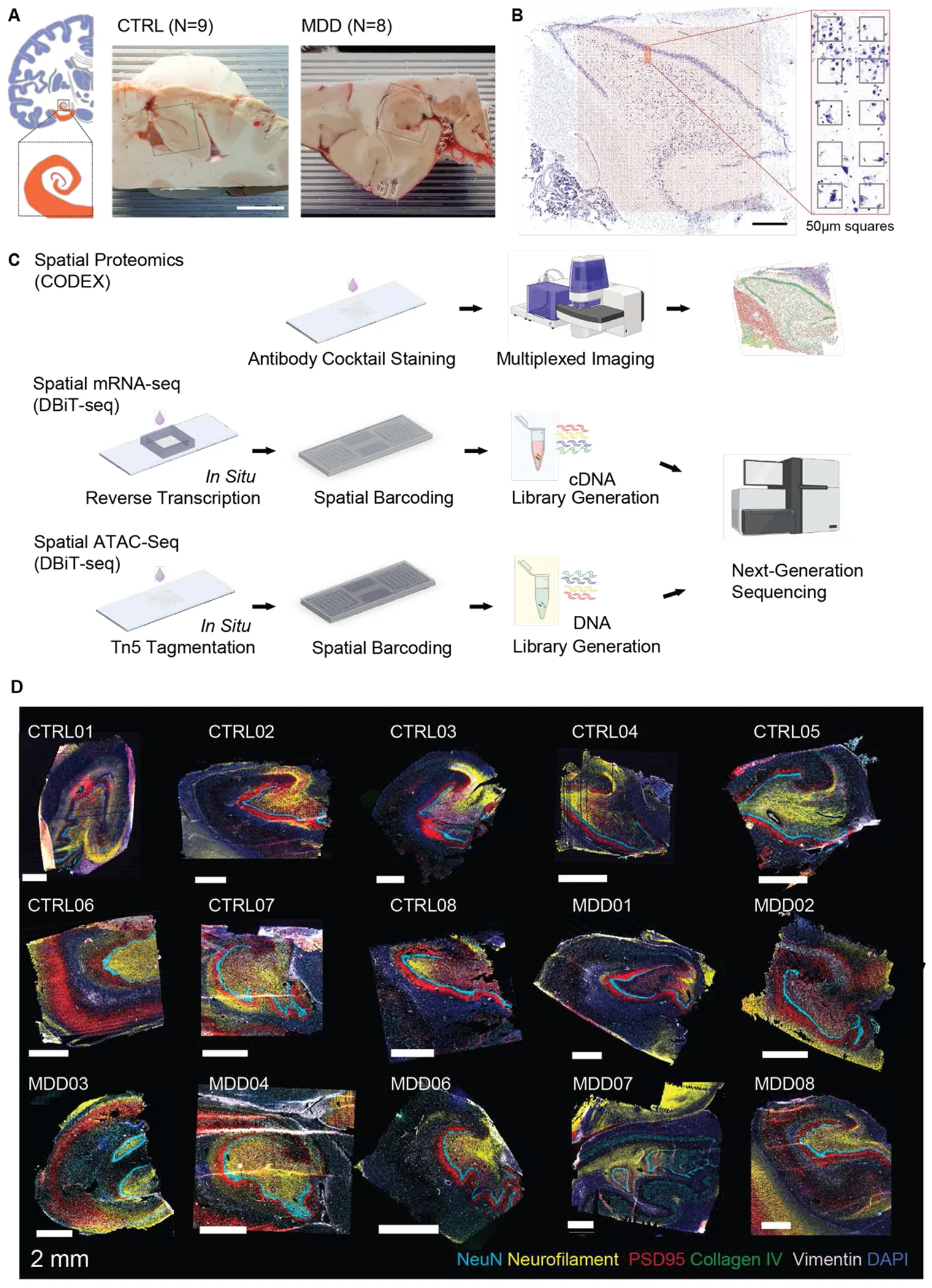
Tissue specimens, spatial omics assays, and global mapping of the human hippocampus. **(A)** Hippocampal regions of interest and representative tissue samples. Scale bar: 10 mm. **(B)** Illustration of the sampling region (5 mm x 5 mm, 2500 sampling spots) and resolution (50 μm) for spatial RNA-seq and ATAC-seq. The representative slice of the human hippocampus was stained with Nissl. Scale bar: 1 mm. **(C)** Schematic of the spatial multi-omics processing workflow. Sequential adjacent tissue sections were profiled with CODEX, RNA-seq, and ATAC-seq. (D) Representative CODEX fluorescence images of 15 human hippocampus. Scale bar: 2 mm.

**Figure 2.**
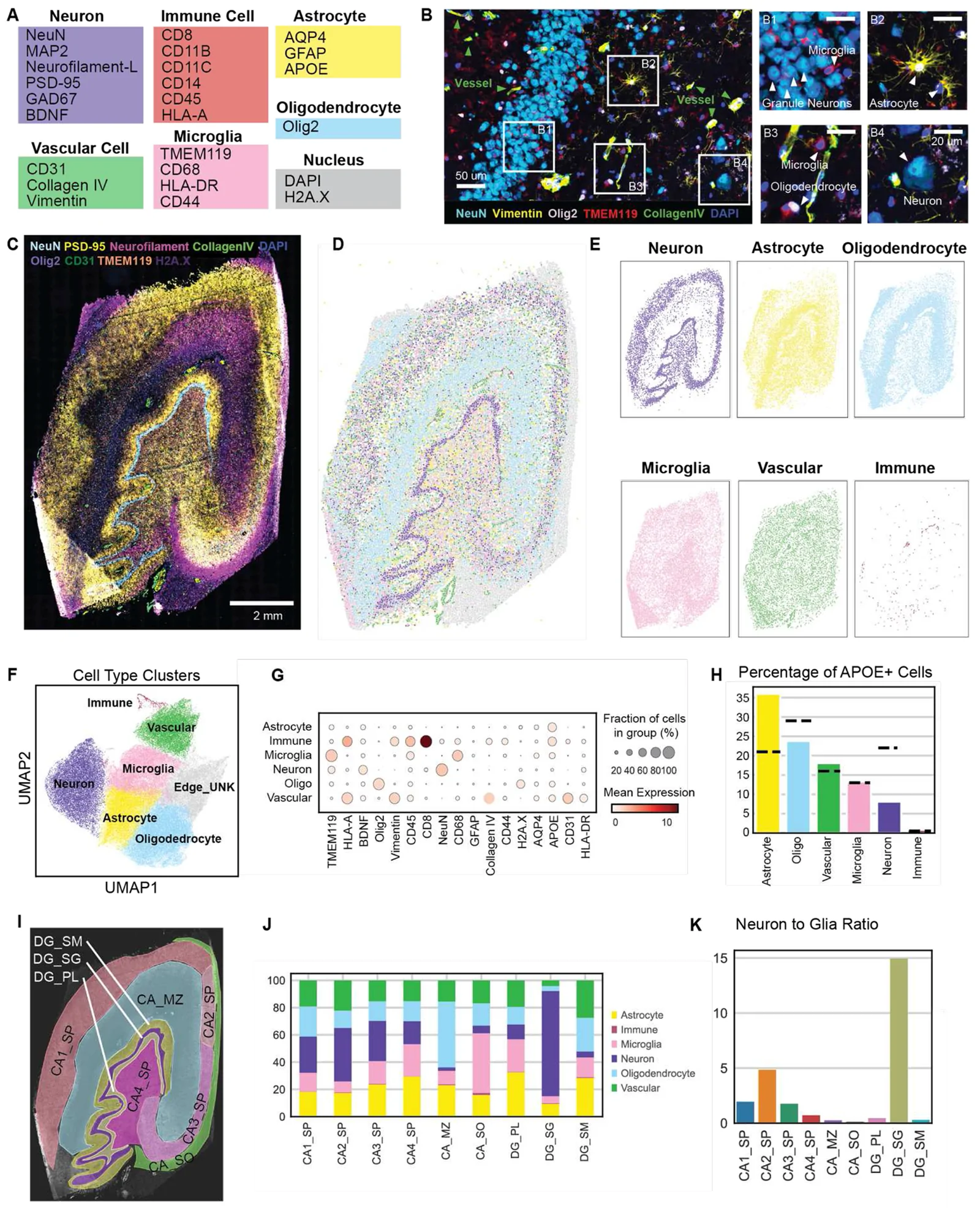
CODEX reveals heterogeneity in hippocampus from neurotypical control at single-cell and functional levels (**A**) The antigens of the 25-antibody panel used to characterize the tissue microenvironment of the human hippocampus. (**B**) A representative region of CODEX fluorescence image of a neurotypical human hippocampus (CTRL01). Six of 25 markers used for the staining are shown: NeuN, a neuron marker (cyan); Vimentin, an astrocyte marker (yellow); Olig2, an oligodendrocyte marker (white); TMEM119, a microglia marker (red); CollagenIV, a blood vessel marker (green); and DAPI, a nuclei marker (blue). Magnified regions showcase diverse range of cell types captured: B1) granule neurons, B2) astrocytes, B3) oligodendrocytes, and B4) mossy cells. Scale bar: 50 μm. (**C**) Full-size CODEX fluorescence image of CTRL01 with five of the 25 markers shown: NeuN (light cyan); PSD95, a synaptic marker enriched in dendrites (yellow); Neurofilament (purple); CollagenIV (green); DAPI (blue); Olig2 (purple); CD31 (dark green); TMEM119 (light orange); and H2A.X(dark purple). Scale bar: 2 mm. (**D**) Full-size image of CRTL01 with cell types mapped to their original tissue coordinates. Cell types are indicated by color: neurons (purple); astrocytes (yellow); oligodendrocytes (cyan); microglia (pink); vascular cells (green); immune cells (dark purple). Each dot represents a single cell. A total of 82,009 cells were identified and assigned with a cell type. (**E**) Images of CRTL01 with individual cell types at their original spatial locations. (**F**) UMAP representation of single-cell CODEX data of CTRL01. Each cluster is colored by its cell-type annotation. The edge and unknown (Edge_UNK) cells are colored gray and were removed prior to downstream analysis. (**G**) Dotplot of key CODEX marker expression for each identified cell type in the CTRL01sample. (**H**) Percentages of APOE^+^ cells per cell type. The dashed line indicates the overall percentage of a given cell type in the entire sample. (**I**) Annotated anatomic hippocampal subfield regions. DG_SG: dentate gyrus stratum granulosum; DG_SM: dentate gyrus stratum moleculare; DG_PL: dentate gyrus polymorphic layer; CA_SP: cornu Ammonis stratum pyramidale; CA_SO: cornu Ammonis stratum oriens: CA_MZ: cornu Ammonis molecular zone. (**J**) Percentages of cell-type compositions for each hippocampal subfield region. X axis shows each hippocampal subfield region. Y axis represents the percentage of each cell type. (**K**) Neuron-to-glia ratio for each hippocampal subfield region. The ratio was computed by dividing the number of neurons by the number of glial cells.

**Figure 3.**
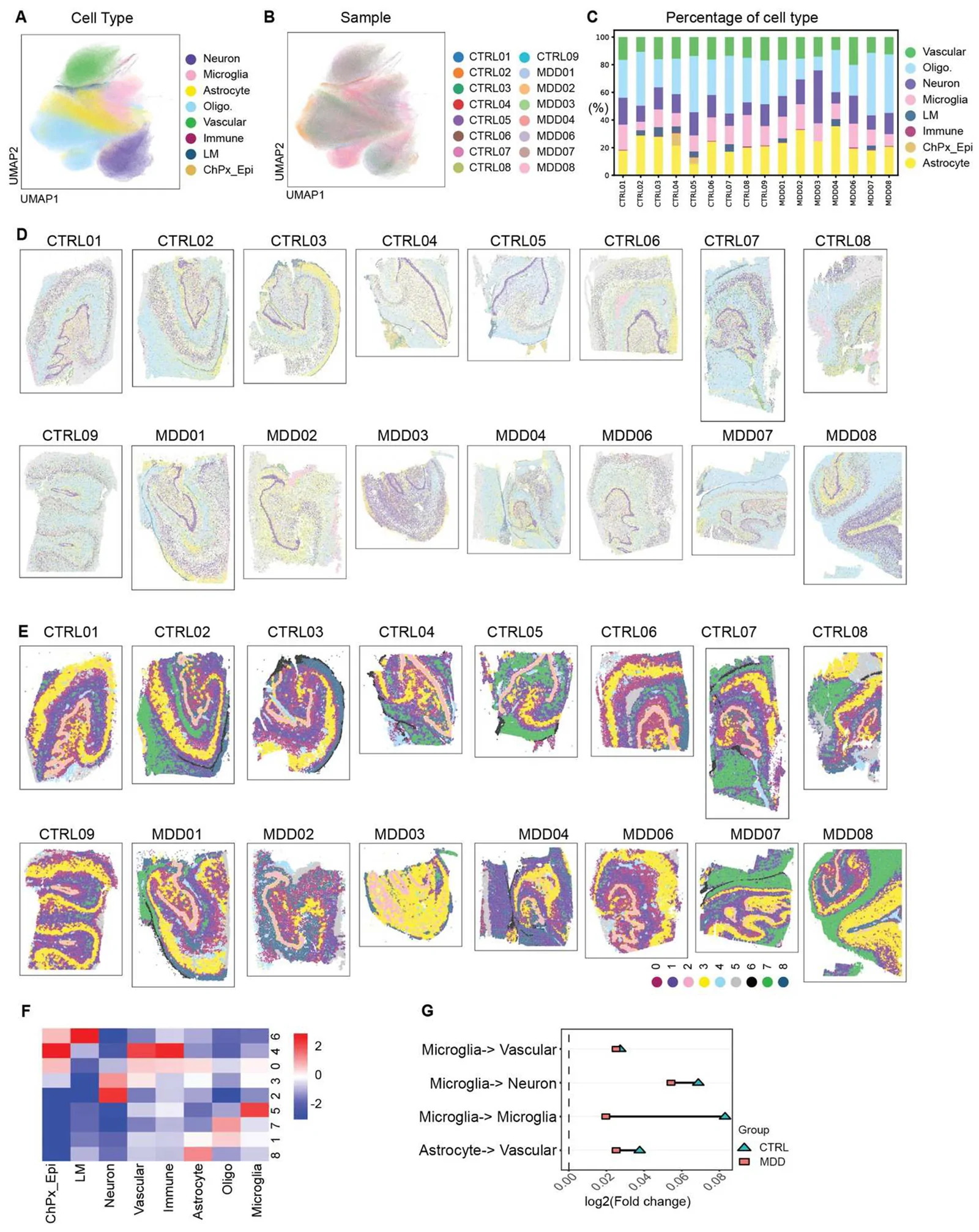
Cellular neighborhoods and cell-cell interactions reveal spatial variation in the hippocampus. (**A**) UMAP representation of all cell types from 16 human hippocampus samples. Each cell is colored according to cell-type annotation: neurons (purple), microglia (pink), astrocytes (yellow), oligodendrocytes (blue), vascular cells (green), immune cells (dark pink), leptomeningeal cells (LM) (dark blue), and choroid plexus epithelial (ChPx_Epi) cells (light brown). (**B**) UMAP representation of all cell types with cells are color-coded based on their sample of origin. (**C**) Percentages of each cell type across the 16 human hippocampus samples. A total of 1,666,549 single cells were profiled and annotated by cell identity. (**D**) Cells colored by type at original tissue coordinates in each sample: neurons (purple), astrocytes (yellow), oligodendrocytes (cyan), microglia (pink), vascular cells (green), and immune cells (dark purple). (**E**) Cellular neighborhoods (CNs) mapped to original tissue coordinates. Each neighborhood corresponds to a known brain anatomic structure. (**F**) Heatmap indicating the enriched cell types within each CN. Values are expressed as Log_2_(Fold Change) (**G**) Selected cell-cell distance pairs. Microglia stayed closer to each other in MDD samples than CTRL samples.

**Figure 4.**
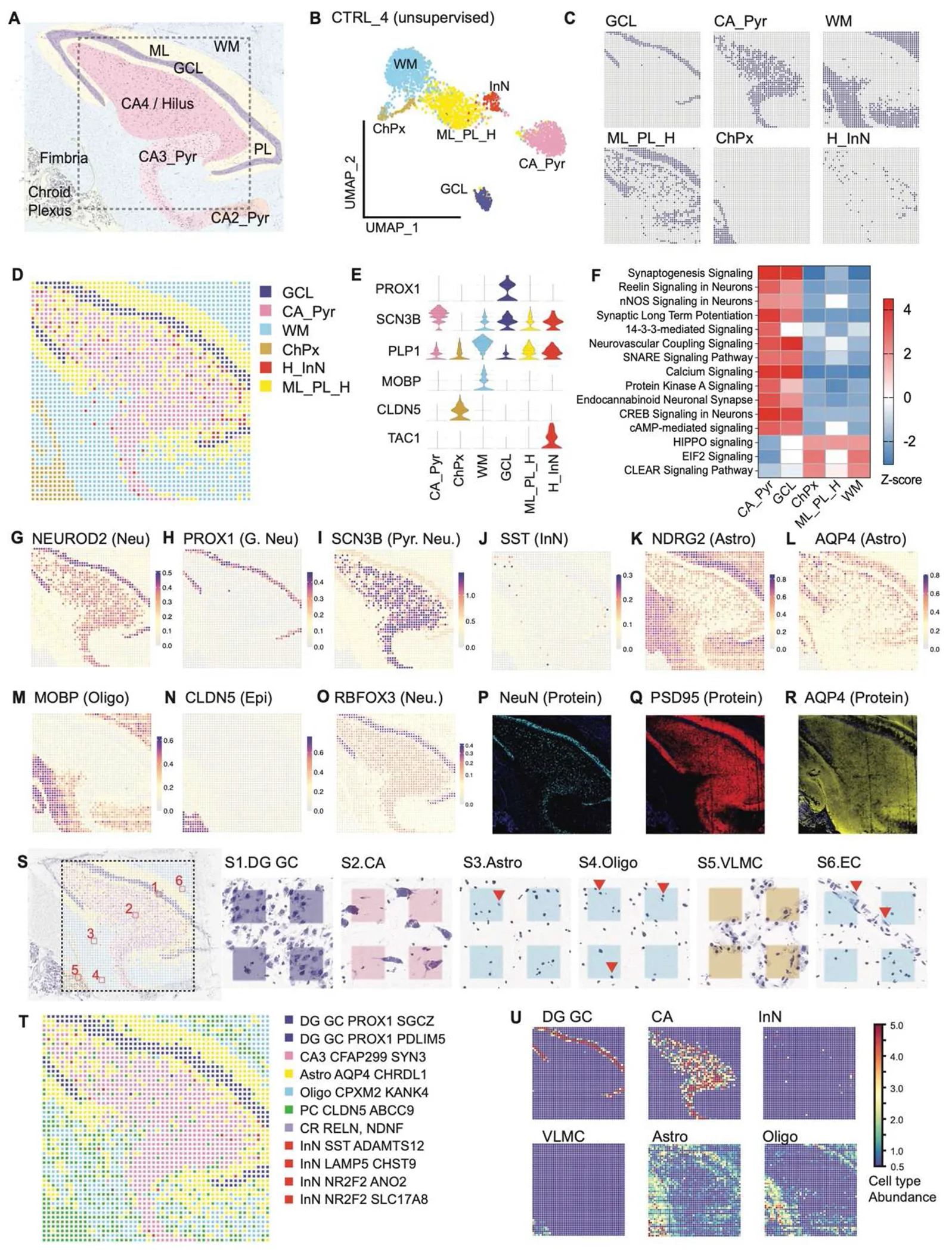
The spatial transcriptome of the neurotypical human hippocampus is heterogeneous. **(A)** Regional annotation of human hippocampus section from CTRL04 based on Nissl stain. The dashed square represented a 5 mm x 5 mm region. GCL: granule cell layer; CA_Pyr: pyramidal neurons in the cornu Ammonis; WM: white matter; ML_PL_H: molecular layer, polymorphic layer, and hilus; ChPx: choroid plexus; InN: inhibitory neurons in the hilus region. **(B)** UMAP plot of spatial transcriptome from CTRL04. Each dot in the UMAP represents the mRNA profile of a single pixel (50 x 50 μm^2^) in the tissue. Clusters correspond to hippocampal subfields. **(C)** Spatial maps showing unsupervised cell clusters in the section from CTRL04. **(D)** Overlay of all cell clusters in one spatial map of section from CTRL04. **(E)** Representative gene markers in each cluster. **(F)** Top canonical pathways in each spatial cluster. **(G-O)** Spatial maps of expression of genes indicative of specific cell types. Neu: neuron; G.Neu: granule neurons; Pyr. Neu.: pyramidal neurons; InN: inhibitory neurons; Astro: astrocytes; Oligo: oligodendrocytes; Micro/Macro: microglia or macrophages; Epi: epithelial cells. **(P-R)** Spatial maps of protein expression determined by CODEX in WHAT samples. NeuN is a neuron-specific protein encoded by *RBFOX3*. PSD95 was used as a marker to visualize dendrites. AQP4 was used as a marker for astrocytes. **(S)** Nissl-stained section of CTRL04 and cell types identified based on morphology. Each spot contained a mixture of cell types in a 50 x 50 μm^2^ sampling region. **(T)** Spatial map of the most abundant cell types in CTRL04 identified by integrative analysis with snRNA-seq data statistically computed by Cell2location algorithm. **(U)** Abundance scores of key cell types in the spatial maps of CTRL04.

**Figure 5.**
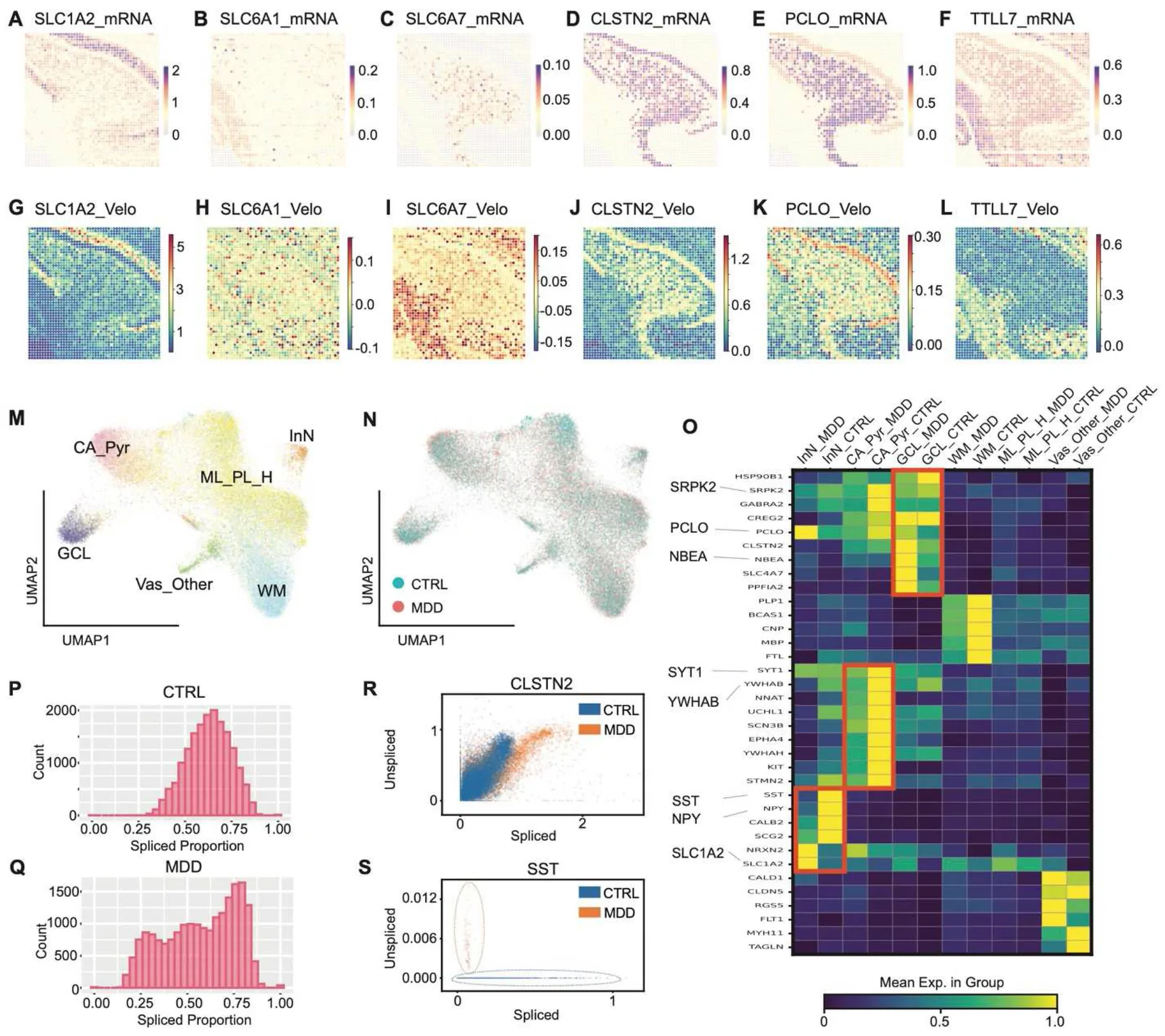
Region-specific gene expression and RNA velocity. **(A-F)** Spatial map of expression of genes related to neurotransmitter transport (SLC1A2, SLC6A1, SLC6A7) and synapse plasticity (CLSTN2, PCLO, TTLL7) in the CTRL04 sample. **(G-L)** Computed RNA velocities of genes related to neurotransmitter transport and synapse plasticity in the CTRL04 sample. Positive values means the RNA level is increasing, negative values means the RNA level is decreasing. Values are normalized across all pixels for each gene. **(M)** Unsupervised clustering of mRNAs in all samples, colored by anatomical regions. Each dot in the UMAP represented a sequencing library collected within a 50 x 50 μm^2^ region. There were 2,500 spots collected from each sample, totaling 37,240 pixels from six control and six MDD samples used for downstream analysis. GCL: Granule Cell Layer; CA_Pyr: pyramidal neurons in the cornu Ammonis; WM: white matter; ML_PL_H: molecular layer, polymorphic layer, and hilus; ChPx: choroid plexus; InN: inhibitory neurons in the hilus region. **(N)** Unsupervised clustering of mRNAs in all samples, colored by disease group. **(O)** Heatmap of highly variable marker expression across clusters. **(P)** The frequency distribution of mature mRNA proportions across all spatial pixels in the CTRL samples (n_CTRL_ = 17500 pixels). **(Q)** The frequency distribution of mature mRNA proportions across all spatial pixels in the MDD samples (n_MDD_ =19740 spatial pixels). **(R)** Phase portrait of the pre- and mature *CLSTN2* mRNAs in all samples. Each point corresponds to a spatial pixel, colored by group (N_total_=11986, n_CTRL_=5006, n_MDD_=6980). **(S)** Phase portrait of pre- and mature *SST* mRNAs in all samples. The cluster circled with orange dashed lines indicates cells from the MDD samples; those circled with blue dashed lines are predominantly from the control samples (N_total_=2085, n_CTRL_=695, n_MDD_=1390).

**Figure 6.**
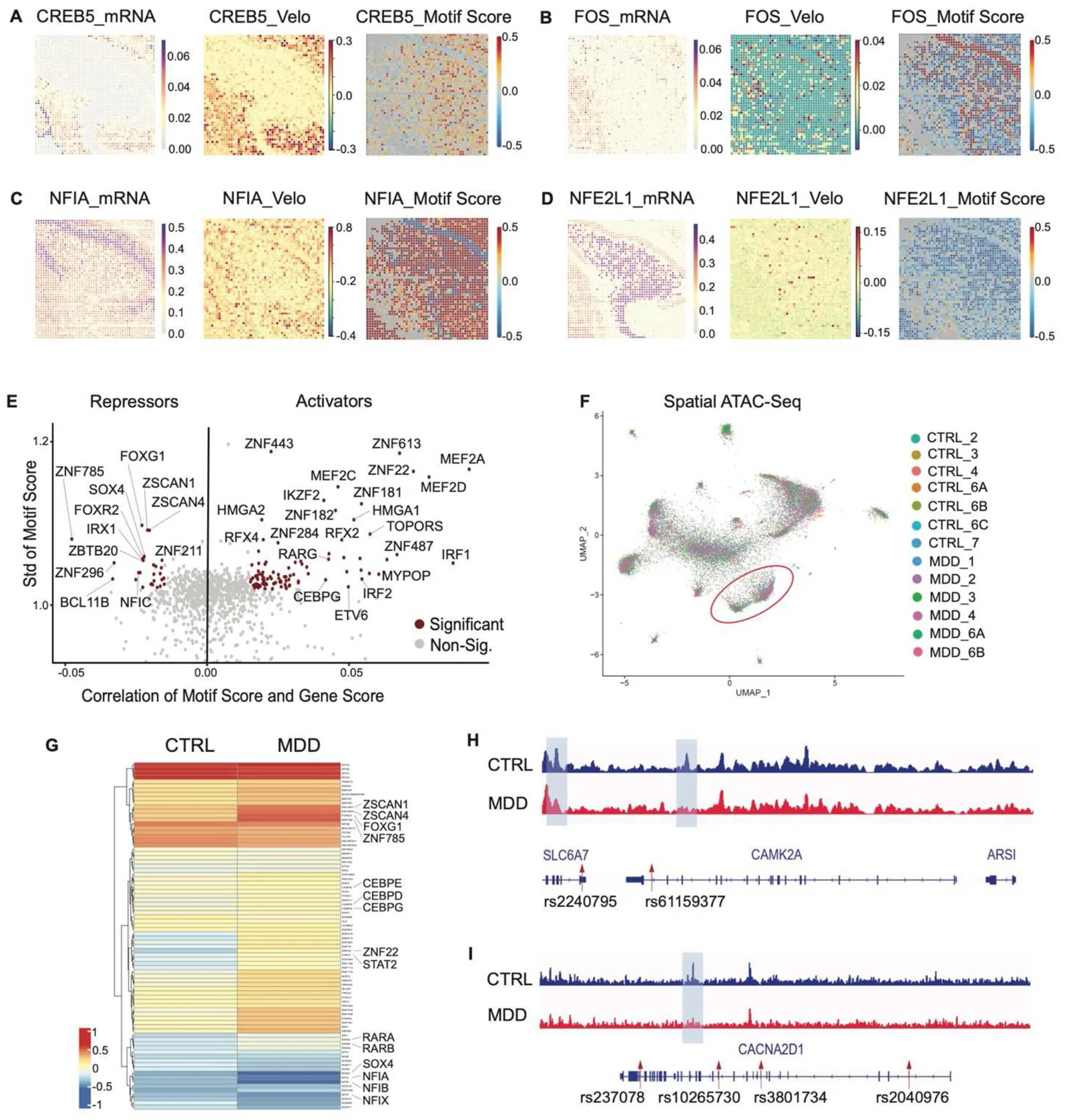
Spatial chromatin accessibility profiling in the dentate gyrus of the hippocampus. **(A-D)** Spatial gene expression, computed RNA velocity based on RNA profiles, and motif scores for select transcription factors [A) CREB5, B) FOS, C) NFIA, D) NFE2L1] in the CTRL04 sample. Spatial mRNA-seq and spatial ATAC-seq were performed on adjacent tissue slices. Gene expression mRNA levels (e.g. CREB5_mRNA) and RNA velocity scores (e.g. CREB5_Velo) were from Spatial mRNA-seq data. The motif scores (e.g. CREB5_Motif Score) were computed based on the cumulative motif sequence reads detected by Spatial ATAC-seq. The motif scores predicted the likelihood of transcription factor binding to specific DNA sequences. **(E)** Correlations of gene expression and transcription factor motif scores across all samples. **(F)** Integrated UMAP embedding of ATAC-seq results for six MDD and seven control samples. Each dot in the UMAP plot represents a sequencing library constructed from the open chromatin collected within a 50 x 50 μm^2^ square of each sample; 2,500 spots were collected from each sample, totaling 32,500 spots. The circled cluster corresponds to GCL regions in all samples. **(G)** Mean motif scores for the most variable transcription factors in GCL of MDD and control samples. **(H-I)** Genome tracks of H) *SLC6A7* and *CAMK2A* and I) *CACNA2D1* in the GCL regions of representative MDD and control samples, highlighting variable gene peaks correlated with known SNPs.

**Table:** Key Resources Table

REAGENT or RESOURCE	SOURCE	IDENTIFIER
Antibody Targets (related to spatial proteomics by CODEX)		
NeuN	Invitrogen	702022
MAP2	Abcam	ab118898
Neurofilament-L	CST	10768SF
PSD-95	Abcam	ab278537
GAD67	Abcam	ab246335
BDNF	Invitrogen	710306
AQP4	CST	59678
APOE	CST	10197
GFAP	Synaptic Systems	173 211
Olig2	Abcam	ab220796
CD31	Akoya Biosciences	AKYP0029
Collagen IV	Akoya Biosciences	AKYP0083
Vimentin	Akoya Biosciences	AKYP0082
TMEM119	CST	83308
CD68	Akoya Biosciences	AKYP0050
HLA-DR	Akoya Biosciences	AKYP0063
CD44	Akoya Biosciences	AKYP0073
CD8	Akoya Biosciences	AKYP0028
CD11B	Abcam	ab187537
CD11C	Akoya Biosciences	AKYP0051
CD14	Akoya Biosciences	AKYP0079
CD45	Akoya Biosciences	AKYP0074
HLA-A	Akoya Biosciences	AKYP0078
γH2A.X	BioLegend	613411
Chemicals, enzymes, and buffers		
20% Paraformaldehyde	Fisher Scientific	50-980-493
DPBS, no calcium, no magnesium	Gibco	14190144
RNA lysis buffer	Qiagen	79216
Triton X-100	Sigma	T8787
Tween-20	Sigma	11332465001
Bovine Serum Albumin (BSA)	Sigma	A8806
Mounting Medium	ThermoFisher	1859332
Maxima H Minus Reverse Transcriptase (200 U/L)	Thermo Fisher Scientific	EP0751
dNTP Mix	Thermo Fisher Scientific	R0192
SUPERase•InTM RNase Inhibitor	Thermo Fisher Scientific	AM2694
DynabeadsTM MyOneTM Streptavidin C1	Thermo Fisher Scientific	65001
Proteinase K, recombinant, PCR grade	Thermo Fisher Scientific	EO0491
RNase Inhibitor	Enzymatics	Y9240L
T4 DNA Ligase	New England Biolabs	M0202L
T4 DNA Ligase Reaction Buffer	New England Biolabs	B0202S
NEBuffer 3.1	New England Biolabs	B7203S
2X NEBNext Master Mix	New England Biolabs	M0541L
Kapa HiFi HotStart ReadyMix PCR Kit	Kapa Biosystems	KK2601
Glycine	Sigma	50046
Tris-HCl (pH 7.4)	Sigma	T2663
NaCl	Thermo Fisher Scientific	AM9760G
MgCl_2_	Thermo Fisher Scientific	AM9530G
Digitonin	Promega	G9441
NP-40	Sigma	11332473001
EDTA solution (pH 8.0)	AmericanBio	AB00502
Tn5 Transposase (unloaded)	Diagenode	C01070010
Tagmentation Buffer (2x)	Diagenode	C01019043
Sodium dodecyl sulfate	Sigma	71736
Ficoll^®^ PM 400	Sigma	F4375
SPRIselect	Beckman Coulter	B23317
EvaGreen^®^ Dye, 20X in Water	Biotium	31000-T
SYBR Green I Nucleic Acid Gel Stain	Thermo Fisher Scientific	S7563
SYLGARDTM 184 Silicone Elastomer Base and Curing Agent	Dow Corning	4019862
Photoresist SU-8 2025	Microchem Laboratory	SU-8 2010
Critical commercial assays and kits		
RNA isolation miRNeasy Micro Kit	Qiagen	1071023
IHC World Nissl Stain Kit	Fisher Scientific	NC0098757
Peroxidase IHC detection kit	Fisher Scientific	PI36000
DNA Clean & Concentrator with Zymo-Spin I Columns	Research Products International	ZD4004
Nextera XT DNA Preparation Kit	Illumina	FC-131-1024
Deposited data		
Sequencing Data		
GSE214144	NCBI GEO	www.ncbi.nlm.nih.gov/geo/query/acc.cgi?acc=GSE214144
Oligonucleotides		
RT Primer	/5Phos/CATCGGCGTACGACTTTTTTTTTTTTTTTTVN	
Primer 1	CAAGCGTTGGCTTCTCGCATCT	
Primer 2	AAGCAGTGGTATCAACGCAGAGT	
Ligation Linker 1	AGTCGTACGCCGATGCGAAACATCGGCCAC	
Ligation Linker 2	CGAATGCTCTGGCCTCTCAAGCACGTGGAT	
Template Switch Primer	AAGCAGTGGTATCAACGCAGAGTGAATrGrG+G	
P5 oligo	AATGATACGGCGACCACCGAGATCTACACTAGATCGCTCGTCGGCAGCGTCAGATGTGTATAAGAGACAG	
P7 oligo (701)	CAAGCAGAAGACGGCATACGAGATTCGCCTTAGTCTCGTGGGCTCGGAGATGTGTATAAGAGACAGCAAGCGTTGGCTTCTCGCATCT	
P7 oligo (702)	CAAGCAGAAGACGGCATACGAGATCTAGTACGGTCTCGTGGGCTCGGAGATGTGTATAAGAGACAGCAAGCGTTGGCTTCTCGCATCT	
P7 oligo (703)	CAAGCAGAAGACGGCATACGAGATTTCTGCCTGTCTCGTGGGCTCGGAGATGTGTATAAGAGACAGCAAGCGTTGGCTTCTCGCATCT	
P7 oligo (704)	CAAGCAGAAGACGGCATACGAGATGCTCAGGAGTCTCGTGGGCTCGGAGATGTGTATAAGAGACAGCAAGCGTTGGCTTCTCGCATCT	
P7 oligo (705)	CAAGCAGAAGACGGCATACGAGATAGGAGTCCGTCTCGTGGGCTCGGAGATGTGTATAAGAGACAGCAAGCGTTGGCTTCTCGCATCT	
P7 oligo (706)	CAAGCAGAAGACGGCATACGAGATCATGCCTAGTCTCGTGGGCTCGGAGATGTGTATAAGAGACAGCAAGCGTTGGCTTCTCGCATCT	
DNA barcodes for spatial location	See Table S2	
Software and algorithms		
Prism (v9.0)	GraphPad Software	N/A
STAR (v2.7.10a)	Ref^65^	https://github.com/alexdobin/STAR
R (v4.1.0)	The R Foundation	https://www.r-project.org
Python (v3.6)	Python Software Foundation	https://www.python.org/
ST-pipeline (v1.80.1)	Ref^66^	https://github.com/jfnavarro/st_pipeline
Seurat (v4.0.4)	Ref^67^	http://satijalab.org/seurat/
Scanpy (v1.9.1)	Ref^56^	https://github.com/scverse/scanpy
Anndata (v0.8.0)	Ref^68^	https://github.com/scverse/anndata
Umap (0.5.3)	Ref ^62^	https://umap-learn.readthedocs.io/
Velocyto (v0.17.16)	Ref^34^	http://velocyto.org/
scVelo (v0.2.1)	Ref^35^	https://scvelo.readthedocs.io/
cell2location	Ref^13^	https://github.com/BayraktarLab/cell2location
ssGSEA	Ref^69^	https://doi.org/10.1016/j.ccell.2017.06.003
ComplexHeatmap (v2.5.6)	Ref^70^	https://jokergoo.github.io/ComplexHeatmap-reference/book/
Ingenuity Pathway Analysis	Qiagen Bioinformatics	https://www.qiagenbioinformatics.com/products/ingenuity-pathway-analysis/
Cell Ranger ATAC (v2.1)	10X Genomics	https://support.10xgenomics.com/single-cell-atac/software/pipelines/latest/what-is-cell-ranger-atac
ArchR (v1.0.1)	Ref^71^	https://www.archrproject.com/
chromVAR	Ref^40^	http://www.github.com/GreenleafLab/chromVAR
MACS2	Ref^63^	https://github.com/macs3-project/MACS
FigR	Ref^64^	https://github.com/buenrostrolab/FigR
Integrative Genomics Viewer (v2.13.2)	Ref^72^	https://www.igv.org/
Tissue Schematics (v1.0.0)	Ref^59^	https://doi.org/10.5281/zenodo.5494009
NeighborhoodCoordination	Ref^58^	https://github.com/nolanlab/NeighborhoodCoordination
MaxFuse	Ref^60^	https://github.com/shuxiaoc/maxfuse/
SPACEc	Ref^29^	https://github.com/yuqiyuqitan/SPACEc
Other		
SuperChipTM Poly-L-Lysine Slides	Electron Microscopy Sciences	63478-AS
Cryostat	Leica	CM3050S
5mm stainless steel bead	Qiagen	69989
Bead mill sample disruptor (TissueLyser II)	Qiagen	85300
RNA Pico Chips	Agilent	5067-1513
Bioanalyzer	Agilent	2100
RNA Screen Tape	Agilent	5067-5576
TapeStation	Agilent	4200
Bright field slide scanner	Leica	SCN400
Inverted microscope	Fisherbrand	03000013
Silicon wafer	WaferPro	C04004
CFX Connect Real-Time PCR Detection System	Bio-Rad	1855201
EVOS^®^ FL Auto Imaging System	Thermo Fisher Scientific	EVOS FL Auto

**Table 1.** Description of postmortem cohort.

Participant	Sex	Age	PMI (Hrs)	GAS	COD	Toxicology	RIN	Source	Proteome	Transcriptome	Epigenome
CTRL01	M	28	12	90	Traffic accident: hemorrhagic shock	Clear	9.40	NYSPI	Y	Y	N
CTRL02	M	27	45.5	N/A	Hypertensive cardiovascular disease	Clear	7.70	NIMH	Y	Y	Y
CTRL03	M	43	15	90	Myocardial Infarction	Clear	8.30	NYSPI	Y	Y	Y
CTRL04	M	31	6.5	90	Industrial accident: traumatic shock	Clear	8.50	NYSPI	Y	Y	Y
CTRL05	M	59	23	91	Accident: traumatic shock	Clear	6.90	NYSPI	Y	Y	N
CTRL06	M	37	9	N/A	Traffic accident: traumatic injuries	Benzodiazepines	6.30	NYSPI	Y	Y	Y
CTRL07	M	39	18	90	Homocide: gun shot wound	Clear	6.70	NYSPI	Y	N	Y
CTRL08	F	44	21	81	Homocide: gun shot wound	Clear	6.00	NYSPI	Y	N	N
CTRL09	M	53	19	90	Hemorrhagic shock ruptured aorta	Clear	7.50	NYSPI	Y	N	N
MDD01	M	42	18	60	Industrial accident: traumatic shock	Clear	7.60	NYSPI	Y	Y	Y
MDD02	M	48	6	50	Suicide: fall from height	Clear	7.90	NYSPI	Y	Y	Y
MDD03	M	47	6	60	Suicide: hanging	Clear	8.30	NYSPI	Y	Y	Y
MDD04	M	53	8	71	Occlusive coronary atherosclerosis	Benzodiazepines	8.40	NYSPI	Y	Y	Y
MDD05	M	66	10	55	Myocardial Infarction	Benzodiazepines Carbon monoxide	7.70	NYSPI	N	Y	N
MDD06	M	30	38	40	Intoxication with insecticide	Methomyl	7.80	NYSPI	Y	Y	Y
MDD07	M	59	11	60	Suicide: hanging	Clear	6.70	NYSPI	Y	N	N
MDD08	F	39	19	70	Suicide: gun shot wound	Benzodiazepines	7.20	NYSPI	Y	N	N

Descriptions of participant samples included in this study. PMI, post-mortem interval; GAS, Global Assessment Scale ^73^; COD, cause of death; RIN, RNA integrity number of the hippocampus; NYSPI: New York State Psychiatric Institute; NIMH: National Institute of Mental Health. The last three columns describe availability of the spatial data reported in this study.

## Data Availability

Spatial mRNA-seq and spatial ATAC-seq data were deposited in the National Center for Biotechnology information Gene Expression Omibus (https://www.ncbi.nlm.nih.gov/geo/) with accession number GSE214144 (Reviewer token: slirmaoqzpuffkt). We did not generate codes for novel algorithms. Custom codes were available upon request without restrictions.

## Highlights

- A spatial atlas of the human hippocampus was created based proteomic, transcriptomic, and epigenomic analyses of neurotypical humans and subjects with major depressive disorder.
- An experimental and computational framework for understanding cellular identity, neighborhood, RNA dynamics, and gene regulatory states with spatial contexts.
- Spatial epigenomic analyses demonstrate that *CREB5* and *FOS* play key roles in neuronal excitability.

## References

[1] Duvernoy H., Cattin F. & Risold P.-Y. in The Human Hippocampus: Functional Anatomy, Vascularization and Serial Sections with MRI (eds Duvernoy Henri M., Francoise Cattin, & Pierre-Yves Risold) 1–237 (Springer, 2013).

[2] Bird C. M. & Burgess N. The hippocampus and memory: insights from spatial processing. Nat Rev Neurosci 9, 182–194 (2008). 10.1038/nrn2335 18270514

[3] Franjic D. Transcriptomic taxonomy and neurogenic trajectories of adult human, macaque, and pig hippocampal and entorhinal cells. Neuron 110, 452–469 e414 (2022). 10.1016/j.neuron.2021.10.036 PMC8813897 34798047

[4] Darmanis S. A survey of human brain transcriptome diversity at the single cell level. Proceedings of the National Academy of Sciences 112, 7285–7290 (2015). 10.1073/pnas.1507125112 PMC4466750 26060301

[5] Ayhan F. Resolving cellular and molecular diversity along the hippocampal anterior-to-posterior axis in humans. Neuron 109, 2091–2105.e2096 (2021). 10.1016/j.neuron.2021.05.003 PMC8273123 34051145

[6] Zhong S. Decoding the development of the human hippocampus. Nature 577, 531–536 (2020). 10.1038/s41586-019-1917-5 31942070

[7] Tran M. N. Single-nucleus transcriptome analysis reveals cell-type-specific molecular signatures across reward circuitry in the human brain. Neuron 109, 3088–3103.e3085 (2021). 10.1016/j.neuron.2021.09.001 PMC8564763 34582785

[8] Skene N. G. Genetic identification of brain cell types underlying schizophrenia. Nat Genet 50, 825–833 (2018). 10.1038/s41588-018-0129-5 PMC6477180 29785013

[9] Moyer C. E., Shelton M. A. & Sweet R. A. Dendritic spine alterations in schizophrenia. Neurosci Lett 601, 46–53 (2015). 10.1016/j.neulet.2014.11.042 PMC4454616 25478958

[10] Amaral D. & Lavenex P. in The Hippocampus Book (eds Andersen P. Ch. 3, 1–168 (Oxford University Press, 2007).

[11] Maynard K. R. Transcriptome-scale spatial gene expression in the human dorsolateral prefrontal cortex. Nat Neurosci 24, 425–436 (2021). 10.1038/s41593-020-00787-0 PMC8095368 33558695

[12] Stickels R. R. Highly sensitive spatial transcriptomics at near-cellular resolution with Slide-seqV2. Nature Biotechnology, 1–7 (2020). 10.1038/s41587-020-0739-1 PMC8606189 33288904

[13] Kleshchevnikov V. Cell2location maps fine-grained cell types in spatial transcriptomics. Nat Biotechnol (2022). 10.1038/s41587-021-01139-4 35027729

[14] Chen W.-T. Spatial Transcriptomics and In Situ Sequencing to Study Alzheimer’s Disease. Cell 182, 976–991 .e919 (2020). 10.1016/j.cell.2020.06.038 32702314

[15] Wang W. Transcriptome dynamics of hippocampal neurogenesis in macaques across the lifespan and aged humans. Cell Res 32, 729–743 (2022). 10.1038/s41422-022-00678-y PMC9343414 35750757

[16] Thompson J. R. An integrated single-nucleus and spatial transcriptomics atlas reveals the molecular landscape of the human hippocampus. Nat Neurosci 28, 1990–2004 (2025). 10.1038/s41593-025-02022-0 PMC12411265 40739059

[17] McEwen B. S. Physiology and neurobiology of stress and adaptation: central role of the brain. Physiol Rev 87, 873–904 (2007). 10.1152/physrev.00041.2006 17615391

[18] Gatt J. M. Interactions between BDNF Val66Met polymorphism and early life stress predict brain and arousal pathways to syndromal depression and anxiety. Molecular Psychiatry 14, 681–695 (2009). 10.1038/mp.2008.143 19153574

[19] Sheline Y. I., Wang P. W., Gado M. H., Csernansky J. G. & Vannier M. W. Hippocampal atrophy in recurrent major depression. Proc Natl Acad Sci U S A 93, 3908–3913 (1996). 10.1073/pnas.93.9.3908 PMC39458 8632988

[20] Malykhin N. V., Carter R., Seres P. & Coupland N. J. Structural changes in the hippocampus in major depressive disorder: contributions of disease and treatment. J Psychiatry Neurosci 35, 337–343 (2010). 10.1503/jpn.100002 PMC2928287 20731966

[21] Cullen K. R. Abnormal amygdala resting-state functional connectivity in adolescent depression. JAMA Psychiatry 71, 1138–1147 (2014). 10.1001/jamapsychiatry.2014.1087 PMC4378862 25133665

[22] Hao Z. Y. Abnormal resting-state functional connectivity of hippocampal subfields in patients with major depressive disorder. BMC Psychiatry 20, 71 (2020). 10.1186/s12888-020-02490-7 PMC7026985 32066415

[23] Sapolsky R. M. Glucocorticoids and hippocampal atrophy in neuropsychiatric disorders. Arch Gen Psychiatry 57, 925–935 (2000). 10.1001/archpsyc.57.10.925 11015810

[24] Deng Y. Spatial profiling of chromatin accessibility in mouse and human tissues. Nature 609, 375–383 (2022). 10.1038/s41586-022-05094-1 PMC9452302 35978191

[25] Kelly T. M. & Mann J. J. Validity of DSM-III-R diagnosis by psychological autopsy: a comparison with clinician ante-mortem diagnosis. Acta Psychiatr.Scand. 94, 337–343 (1996). 10.1111/j.1600-0447.1996.tb09869.x 9124080

[26] Goltsev Y. Deep Profiling of Mouse Splenic Architecture with CODEX Multiplexed Imaging. Cell 174, 968–981 e915 (2018). 10.1016/j.cell.2018.07.010 PMC6086938 30078711

[27] Hickey J. W. Organization of the human intestine at single-cell resolution. Nature 619, 572–584 (2023). 10.1038/s41586-023-05915-x PMC10356619 37468586

[28] Liao F., Yoon H. & Kim J. Apolipoprotein E metabolism and functions in brain and its role in Alzheimer’s disease. Curr Opin Lipidol 28, 60–67 (2017). 10.1097/MOL.0000000000000383 PMC5213812 27922847

[29] Tan Y. SPACEc: a streamlined, interactive Python workflow for multiplexed image processing and analysis. Nat Commun 16, 10652 (2025). 10.1038/s41467-025-65658-3 PMC12660697 41309581

[30] Liu Y. High-Spatial-Resolution Multi-Omics Sequencing via Deterministic Barcoding in Tissue. Cell 183, 1665–1681 e1618 (2020). 10.1016/j.cell.2020.10.026 PMC7736559 33188776

[31] Su G. Spatial multi-omics sequencing for fixed tissue via DBiT-seq. STAR Protoc 2, 100532 (2021). 10.1016/j.xpro.2021.100532 PMC8132129 34027489

[32] Pelkey K. A. Hippocampal GABAergic Inhibitory Interneurons. Physiol Rev 97, 1619–1747 (2017). 10.1152/physrev.00007.2017 PMC6151493 28954853

[33] García-Cabezas M., John Y. J., Barbas H. & Zikopoulos B. Distinction of Neurons, Glia and Endothelial Cells in the Cerebral Cortex: An Algorithm Based on Cytological Features. Frontiers in neuroanatomy 10, 107 (2016). 10.3389/fnana.2016.00107 PMC5088408 27847469

[34] La Manno G. RNA velocity of single cells. Nature 560, 494–498 (2018). 10.1038/s41586-018-0414-6 PMC6130801 30089906

[35] Bergen V, Lange M., Peidli S., Wolf F. A. & Theis F. J. Generalizing RNA velocity to transient cell states through dynamical modeling. Nature Biotechnology, 1–7 (2020). 10.1038/s41587-020-0591-3 32747759

[36] Barnat M. The GSK3-MAP1B pathway controls neurite branching and microtubule dynamics. Molecular and cellular neurosciences 72, 9–21 (2016). 10.1016/j.mcn.2016.01.001 26773468

[37] Dhindsa R. S., Lowenstein D. H. & Goldstein D. B. in Genomics, Circuits, and Pathways in Clinical Neuropsychiatry (eds Lehner Thomas, Miller Bruce L., & State Matthew W.) 601–617 (Academic Press, 2016).

[38] Minelli A. PCLO gene: its role in vulnerability to major depressive disorder. J Affect Disord 139, 250–255 (2012). 10.1016/j.jad.2012.01.028 22386049

[39] Maddox D. M. A Mutation in Syne2 Causes Early Retinal Defects in Photoreceptors, Secondary Neurons, and Muller Glia. Invest Ophthalmol Vis Sci 56, 3776–3787 (2015). 10.1167/iovs.14-16047 PMC4468423 26066746

[40] Schep A. N., Wu B., Buenrostro J. D. & Greenleaf W. J. chromVAR: inferring transcription-factor-associated accessibility from single-cell epigenomic data. Nat Methods 14, 975–978 (2017). 10.1038/nmeth.4401 PMC5623146 28825706

[41] Kandel E. R. The molecular biology of memory: cAMP, PKA, CRE, CREB-1, CREB-2, and CPEB. Mol Brain 5, 14 (2012). 10.1186/1756-6606-5-14 PMC3514210 22583753

[42] Minatohara K., Akiyoshi M. & Okuno H. Role of Immediate-Early Genes in Synaptic Plasticity and Neuronal Ensembles Underlying the Memory Trace. Front Mol Neurosci 8, 78 (2015). 10.3389/fnmol.2015.00078 PMC4700275 26778955

[43] Liu X. Optogenetic stimulation of a hippocampal engram activates fear memory recall. Nature 484, 381–385 (2012). 10.1038/nature11028 PMC3331914 22441246

[44] Huang A. Y. Region-Specific Transcriptional Control of Astrocyte Function Oversees Local Circuit Activities. Neuron 106, 992–1008 e1009 (2020). 10.1016/j.neuron.2020.03.025 PMC7879989 32320644

[45] Luczynska K., Zhang Z., Pietras T., Zhang Y. & Taniguchi H. NFE2L1/Nrf1 serves as a potential therapeutical target for neurodegenerative diseases. Redox Biol 69, 103003 (2024). 10.1016/j.redox.2023.103003 PMC10788251 38150994

[46] Yiu A. P. Neurons are recruited to a memory trace based on relative neuronal excitability immediately before training. Neuron 83, 722–735 (2014). 10.1016/j.neuron.2014.07.017 25102562

[47] LaFave L. M. Epigenomic State Transitions Characterize Tumor Progression in Mouse Lung Adenocarcinoma. Cancer Cell 38, 212–228 e213 (2020). 10.1016/j.ccell.2020.06.006 PMC7641015 32707078

[48] Li L., Rispoli R., Patient R., Ciau-Uitz A. & Porcher C. Etv6 activates vegfa expression through positive and negative transcriptional regulatory networks in Xenopus embryos. Nat Commun 10, 1083 (2019). 10.1038/s41467-019-09050-y PMC6403364 30842454

[49] Wray N. R. Genome-wide association analyses identify 44 risk variants and refine the genetic architecture of major depression. Nat Genet 50, 668–681 (2018). 10.1038/s41588-018-0090-3 PMC5934326 29700475

[50] Turecki G. & Meaney M. J. Effects of the Social Environment and Stress on Glucocorticoid Receptor Gene Methylation: A Systematic Review. Biol Psychiatry 79, 87–96 (2016). 10.1016/j.biopsych.2014.11.022 PMC4466091 25687413

[51] Smith K. E. & Pollak S. D. Early life stress and development: potential mechanisms for adverse outcomes. J Neurodev Disord 12, 34 (2020). 10.1186/s11689-020-09337-y PMC7745388 33327939

[52] Rosoklija G., Duma A. & Dwork A. J. Psychiatric brain collection in Macedonia: general lessons for scientific collaboration among countries of differing wealth. Pril (Makedon Akad Nauk Umet Odd Med Nauki) 34, 95–98 (2013). PMC4086878 23917747

[53] Tan Y. SPACEc: A Streamlined, Interactive Python Workflow for Multiplexed Image Processing and Analysis. bioRxiv, 2024.2006.2029.601349 (2024). 10.1101/2024.06.29.601349 PMC12660697 41309581

[54] Lee M. Y. CellSeg: a robust, pre-trained nucleus segmentation and pixel quantification software for highly multiplexed fluorescence images. BMC Bioinformatics 23, 46 (2022). 10.1186/s12859-022-04570-9 PMC8767664 35042474

[55] Hickey J. W., Tan Y., Nolan G. P. & Goltsev Y. Strategies for Accurate Cell Type Identification in CODEX Multiplexed Imaging Data. Front Immunol 12, 727626 (2021). 10.3389/fimmu.2021.727626 PMC8415085 34484237

[56] Wolf F. A., Angerer P. & Theis F. J. SCANPY: large-scale single-cell gene expression data analysis. Genome Biol 19, 15 (2018). 10.1186/s13059-017-1382-0 PMC5802054 29409532

[57] Jiang S. Combined protein and nucleic acid imaging reveals virus-dependent B cell and macrophage immunosuppression of tissue microenvironments. Immunity 55, 1118–1134 e1118 (2022). 10.1016/j.immuni.2022.03.020 PMC9220319 35447093

[58] Schurch C. M. Coordinated Cellular Neighborhoods Orchestrate Antitumoral Immunity at the Colorectal Cancer Invasive Front. Cell 182, 1341–1359 e1319 (2020). 10.1016/j.cell.2020.07.005 PMC7479520 32763154

[59] Bhate S. S., Barlow G. L., Schurch C. M. & Nolan G. P. Tissue schematics map the specialization of immune tissue motifs and their appropriation by tumors. Cell Syst 13, 109–130 e106 (2022). 10.1016/j.cels.2021.09.012 PMC12588373 34653369

[60] Chen S. Integration of spatial and single-cell data across modalities with weakly linked features. Nat Biotechnol (2023). 10.1038/s41587-023-01935-0 PMC11638971 37679544

[61] Benitez A. D. N. Spatial and simultaneous representative seroprevalence of anti-Toxoplasma gondii antibodies in owners and their domiciled dogs in a major city of southern Brazil. PLoS One 12, e0180906 (2017). 10.1371/journal.pone.0180906 PMC5521765 28732033

[62] McInnes L., Healy J. & Melville J. UMAP: Uniform Manifold Approximation and Projection for Dimension Reduction. arXiv:1802.03426 [cs, stat] (2020).

[63] Zhang Y. Model-based analysis of ChIP-Seq (MACS). Genome Biol 9, R137 (2008). 10.1186/gb-2008-9-9-r137 PMC2592715 18798982

[64] Kartha V. K. Functional inference of gene regulation using single-cell multi-omics. Cell Genomics, 100166 (2022). 10.1016/j.xgen.2022.100166 PMC9534481 36204155

[65] Dobin A. STAR: ultrafast universal RNA-seq aligner. Bioinformatics 29, 15–21 (2013). 10.1093/bioinformatics/bts635 PMC3530905 23104886

[66] Navarro J. F., Sjostrand J., Salmen F., Lundeberg J. & Stahl P. L. ST Pipeline: an automated pipeline for spatial mapping of unique transcripts. Bioinformatics 33, 2591–2593 (2017). 10.1093/bioinformatics/btx211 28398467

[67] Satija R., Farrell J. A., Gennert D., Schier A. F. & Regev A. Spatial reconstruction of single-cell gene expression data. Nat Biotechnol 33, 495–502 (2015). 10.1038/nbt.3192 PMC4430369 25867923

[68] Virshup I., Rybakov S., Theis F. J., Angerer P. & Wolf F. A. anndata: Annotated data. bioRxiv, 2021.2012.2016.473007 (2021). 10.1101/2021.12.16.473007

[69] Wang Q. Tumor Evolution of Glioma-Intrinsic Gene Expression Subtypes Associates with Immunological Changes in the Microenvironment. Cancer Cell 32, 42–56 e46 (2017). 10.1016/j.ccell.2017.06.003 PMC5599156 28697342

[70] Gu Z., Eils R. & Schlesner M. Complex heatmaps reveal patterns and correlations in multidimensional genomic data. Bioinformatics 32, 2847–2849 (2016). 10.1093/bioinformatics/btw313 27207943

[71] Granja J. M. ArchR is a scalable software package for integrative single-cell chromatin accessibility analysis. Nat Genet 53, 403–411 (2021). 10.1038/s41588-021-00790-6 PMC8012210 33633365

[72] Robinson J. T. Integrative genomics viewer. Nat Biotechnol 29, 24–26 (2011). 10.1038/nbt.1754 PMC3346182 21221095

[73] Endicott J., Spitzer R. L., Fleiss J. L. & Cohen J. The global assessment scale. A procedure for measuring overall severity of psychiatric disturbance. Arch Gen Psychiatry 33, 766–771 (1976). 10.1001/archpsyc.1976.01770060086012 938196

